# Copper-promoted hydration and annulation of 2-fluorophenylacetylene derivatives: from alkynes to benzo[*b*]furans and benzo[*b*]thiophenes

**DOI:** 10.3762/bjoc.10.305

**Published:** 2014-12-04

**Authors:** Yibiao Li, Liang Cheng, Xiaohang Liu, Bin Li, Ning Sun

**Affiliations:** 1 School of Chemical & Environmental Engineering, Wuyi University, Jiangmen, Guangdong Province, 529090, China; 2 BASF Catalyst, 23800 Mercantile Road, Beachwood, Ohio 44124, USA

**Keywords:** annulation, benzo[*b*]furan, C–F activation, copper-promoted, heterocycle

## Abstract

An efficient copper-promoted hydration reaction and its application in the synthesis of benzo[*b*]furan and benzo[*b*]thiophene derivatives is presented starting from readily available 2-fluorophenylacetylene derivatives. The key annulation step involves the hydration of the C–F bond of 2-fluorophenylacetylene derivatives followed by an intramolecular annulation to afford benzo[*b*]furan and benzo[*b*]thiophene derivatives. Moreover, structurally important 2,2'-bisbenzofuran scaffolds are provided in good yields.

## Introduction

**Scheme 1:**
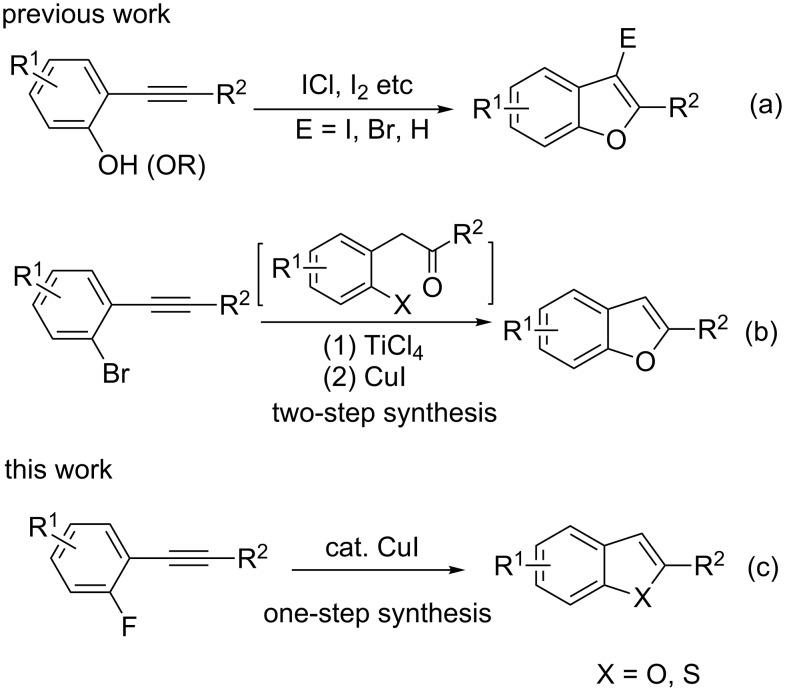
Synthetic approaches to benzo[*b*]furans from 2-alkynylphenols, ketones and 2-fluorophenylacetylene derivatives.

The development of general and efficient methodologies for the synthesis of complex heterocycle skeletons has received much attention in the past decades. Among the most ubiquitous heterocyclic moieties in natural and bioactive products are the benzo[*b*]furan and benzo[*b*]thiophene units [1–8]. Despite the existence of established methods for the synthesis of benzo[*b*]furan and benzo[*b*]thiophene derivatives, the development of more convenient methods is of significant importance [9–14]. Commonly, the preparation of 2-substituted benzo[*b*]furans involves the usage of 2-halophenols as reaction precursors (Scheme 1a) [15–18], which can be cumbersome due to the precursors’ instability and the protecting and deprotecting steps necessary to synthesize the precursors [19–23]. Ackermann et al. utilized bromo- and iodo-substituted phenylacetylene in their TiCl_4_-catalyzed intramolecular nucleophilic annulation process (Scheme 1b) [24]. But this method involves a two-step process and the usage of two different metal salts may complicate further processing. The direct design of a Pd or Cu-catalyzed one-pot synthesis of benzo[*b*]thiophenes from 2-bromoalkynylbenzenes and a thiol derivative has eliminated these problems to a large extent [25–29]. Nevertheless, the direct synthesis of benzo[*b*]furans from 2-haloalkynylbenzenes and the usage of 2-fluorophenylacetylene derivatives as substrates continues to represent a challenge. Indeed, Tsuji and co-workers have developed a transition metal-free process for the synthesis of benzo[*b*]furans from 2-fluorophenylacetylene derivatives. But the reaction requires conditions with a high reaction temperature for satisfactory yields. Unfortunately, only benzo[*b*]furans were obtained in this reaction [30].

Typically, the aryl halides used in the annulation reactions are iodides and bromides. It is rare to employ aryl fluorides because of their low reactivity [31–33]. To extend the application of our strategy of the copper-catalyzed synthesis of heterocycles, we report herein a one-pot process for the synthesis of benzo[*b*]furans and benzo[*b*]thiophenes with 2-fluorophenylacetylene derivatives as precursors (Scheme 1c).

## Results and Discussion

We report an efficient synthesis of functionalized benzo[*b*]furans from commercially available alkynes by a copper-catalyzed, intramolecular annulation process. Initially, our investigation commenced with the annulation of (2-(2-fluorophenyl)ethynyl)benzene (**1a**) to give the corresponding product 2-phenylbenzofuran (**2a**) by using 2 equiv KOH as a base under various conditions. In the presence of the Pd(PPh_3_)_4_ catalyst the reaction of (2-(2-fluorophenyl)ethynyl)benzene in CH_3_CN does not give any corresponding product (Table 1, entry 1). The usage of CuCl and 1,10-phenanthroline (1,10-phen) as a ligand in CH_3_CN at 80 °C showed that **2a** could be isolated in 35% yield (Table 1, entry 2). The screening of the various solvents revealed that the solvent played an important role in this hydration and annulation process. Compared with the other solvents, DMSO is more suitable for the annulation process (Table 1, entries 2–4). These investigations revealed that the usage of CuI instead of CuCl as a catalyst resulted in the isolation of **2a** in a satisfactory 88% yield after 4 hour (Table 1, entry 5). To our delight, the use of 0.2 equiv of KI as an additive afforded **2a** in a satisfactory 95% yield (Table 1, entry 6). The base loading had a strong influence on the yield with 2 equiv KOH being the optimal amount (Table 1, entries 7 and 12). Further screening of bases did not lead to better yields and confirmed that the reaction did not proceed in the presence of CsCO_3_ (Table 1, entry 8). Other catalytic systems, such as Cu(OAc)_2_, Cu(OTf)_2_ and Cu(acac)_2_, were less effective for this annulation process (Table 1, entries 9–11). A decrease in the temperature lowered the yield of the reaction (Table 1, entry 13). The importance of water was confirmed by a lower yield under dry conditions (Table 1, entry 14). In the absence of CuI, we found that the reaction of (2-(2-fluorophenyl)ethynyl)benzene with KOH in DMSO at 80 °C for 4 h gave 55% yield of the annulation product (Table 1, entry 15).

**Table 1:** Optimization of the reaction conditions.^a^

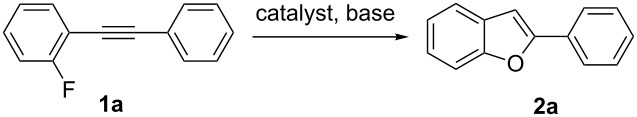				

Entry	Catalyst	Solvent	Additive	Yield [%]^b^

1	Pd(PPh_3_)_4_	CH_3_CN	–	–
2	CuCl	CH_3_CN	1,10-phen	35
3	CuCl	DMF	1,10-phen	71
4	CuCl	DMSO	1,10-phen	75
5	CuI	DMSO	1,10-phen	88
6	CuI	DMSO	KI	95
7^c^	CuI	DMSO	KI	68
8^d^	CuI	DMSO	KI	<5
9	Cu(OAc)_2_	DMSO	KI	65
10	Cu(OTf)_2_	DMSO	KI	68
11	Cu(acac)_2_	DMSO	KI	54
12^e^	CuI	DMSO	KI	<5
13^f^	CuI	DMSO	KI	25
14^g^	CuI	DMSO	KI	38
15	–	DMSO	KI	55

^a^Reaction conditions: alkyne **1a** (1.0 mmol), catalyst (10 mol %), base (2.0 mmol), H_2_O (1.5 mmol) and additives (0.2 mmol) in 3 mL of solvent at 80 °C for 4 h; ^b^yields are given for isolated products; ^c^1 equiv KOH was used; ^d^CsCO_3_ instead of KOH; ^e^omitting KOH and starting material recovered; ^f^reaction was carried out at 30 °C. ^g^omitting H_2_O (dry conditions).

**Scheme 2:**
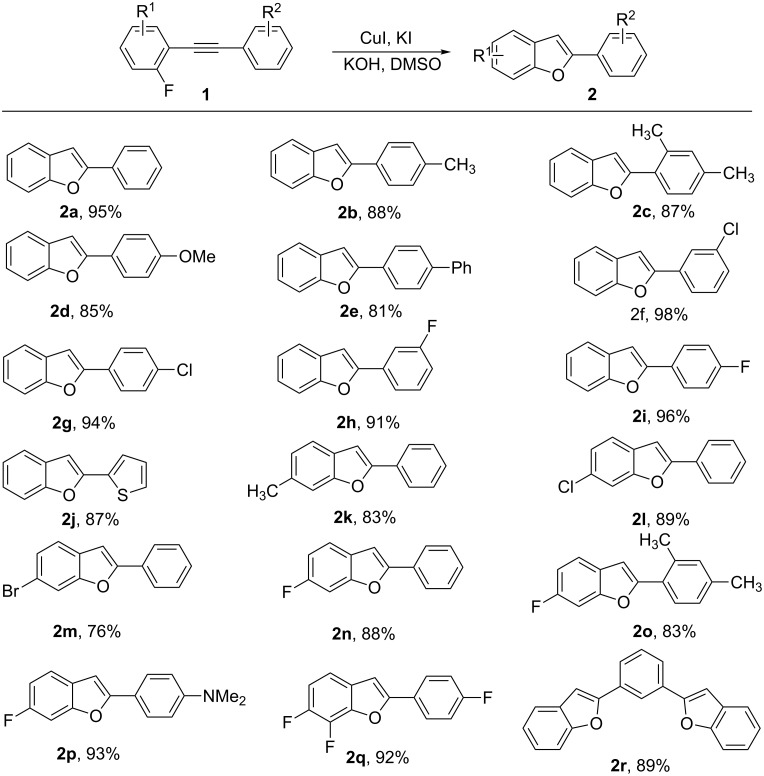
Copper-promoted reaction of 2-fluorophenylacetylene derivatives to yield benzo[*b*]furans. Reaction conditions: Alkyne **1a** (1.0 mmol), catalyst (10 mol %), KOH (2.0 mmol), H_2_O (1.5 mmol) and KI (0.2 mmol) in 3 mL of DMSO at 80 °C for 4–8 h; yields are given for isolated products.

Next, we explored the scope and generality of the process by using the conditions for Tabe 1, entry 6. As shown in Scheme 2, substrates with either electron-donating or electron-withdrawing substituents on the benzene ring can undergo the reaction smoothly, and the corresponding benzo[*b*]furan products were obtained in good to excellent yields. The reaction tolerated a variety of substituents including -Cl, -Br, -F, -OMe, -NMe_2_ and thiophenyl groups. The use of 2-fluorophenylacetylene derivatives with electron-withdrawing substituents as R^2^ afforded benzo[*b*]furan products in higher yields. It is noteworthy that the 2-(2-(2-fluorophenyl)ethynyl)thiophene was also successfully converted to 2-(thiophen-2-yl)benzofuran (**2j**) in good yields. Subsequently, the R^1^ substituent of the 2-fluorophenylacetylene derivatives was varied from hydrogen to other functional groups. Substituents at the ortho position of the benzyl group did not have an impact on the reaction yield. The presence of an additional electron-donating substituent marginally decreased the conversion of 2-fluorophenylacetylene derivatives resulting in products in moderate yields (Scheme 2, **2k** and **2o**). Interestingly, the *p*-fluoro atom was kept intact during the reaction and fluoro-substituted benzofuran was obtained (Scheme 2, **2q**) [34–37]. This shows the good selectivity of the current reaction system. It should be emphasized that the 1,3-bis(2-(2-fluorophenyl)ethynyl)benzene was also successfully converted to benzo[*b*]furan **2r** in good yield. Unfortunately, when aliphatic alkynes were employed, the desired annulation products were formed in low yields.

**Scheme 3:**
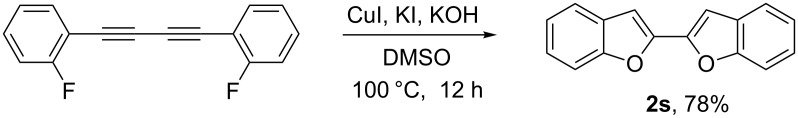
Copper-promoted synthesis of 2,2'-bisbenzofuran derivatives.

The above studies dealt only with 1,2-diphenylethyne derivatives as a reactive group in the substrates. Inspired by the results of the nucleophilic annulation process, we wondered whether we could further explore the annulation of 1,3-diynes, which have great synthetic potential in medicine and materials sciences [38–40]. For extensions, we used 1,4-bis(2-fluorophenyl)buta-1,3-diyne as a substrate to investigate the possibility of this transformation. Similar to (2-(2-fluorophenyl)ethynyl)benzene, 1,4-bis(2-fluorophenyl)buta-1,3-diyne was able to offer the corresponding annulation products **2s** in 78% yield (Scheme 3).

**Scheme 4:**
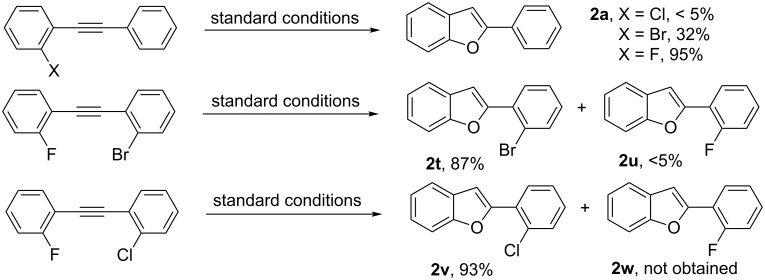
Intramolecular competition experiments.

To gain a deeper mechanistic understanding of the present catalytic process, the direct intramolecular annulation of 1-bromo-2-(2-(2-fluorophenyl)ethynyl)benzene and 1-chloro-2-(2-(2-fluorophenyl)ethynyl)benzene were performed, as shown in Scheme 4. In F/Br-substituted 1-bromo-2-(2-(2-fluorophenyl)ethynyl)benzene, the fluoro moiety served as leaving group and gave 2-(2-bromophenyl)benzofuran (**2t**) as a major product. In F/Cl-substituted 1-chloro-2-(2-(2-fluorophenyl)ethynyl)benzene was able to offer the chloro-substituted product **2v** as the only product. A reactivity order of F > Br > Cl can be derived from these data.

**Scheme 5:**
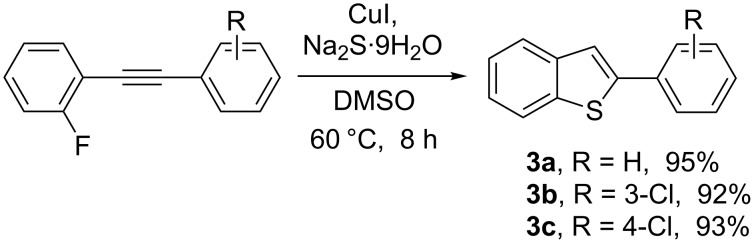
Copper-promoted synthesis of benzo[*b*]thiophenes.

Reactions with Na_2_S·9H_2_O as a nucleophile were successful, and the corresponding benzo[*b*]thiophene products were obtained in high yields (Scheme 5). We obtained the best results with DMSO as the solvent and a reaction temperature of 60 °C. Using the optimized reaction conditions, 3-chloro and 4-chloro substituted 2-fluoroalkynylbenzenes were reacted with Na_2_S·9H_2_O to yield benzo[*b*]thiophenes in good yields.

**Scheme 6:**
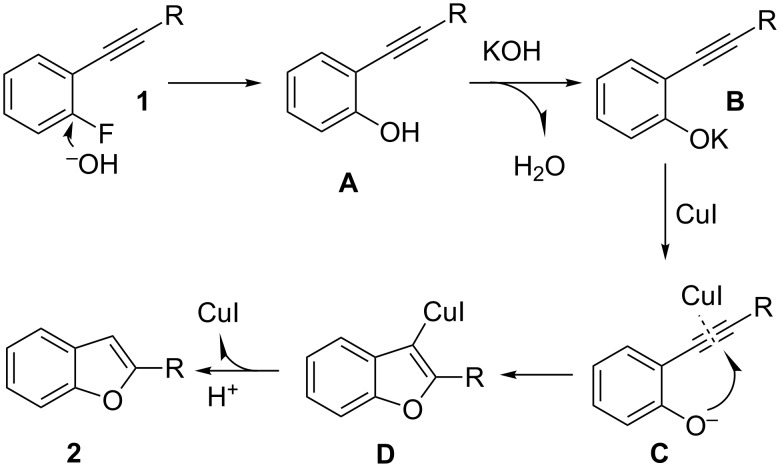
Proposed mechanism for the annulation reaction.

The postulated reaction mechanism is depicted in Scheme 6 [25–29]. The catalytic cycle is initiated by the nucleophilic substitution of 2-fluorophenylacetylene derivative **1** with OH^−^. This might provide unstable 2-alkynylphenol **A**, which could then form the corresponding potassium phenolate intermediate **B**. The coordination of CuI with **B** may provide intermediate **C**, and the subsequent addition to the C–C triple bond gives the copper complex **D**. Protonolysis of intermediate **D** generates benzo[*b*]furan **2** and regenerates the active catalyst species.

## Conclusion

In summary, we have developed a new protocol for the synthesis of benzo[*b*]furan and benzo[*b*]thiophene derivatives starting from 2-fluorophenylacetylene derivatives. The hydration and annulation is catalyzed by CuI with KOH or Na_2_S·9H_2_O as a base at 60–80 °C to give the corresponding products in moderate to good yields. Various functional groups are accepted resulting in a wide range of substituted benzo[*b*]furans and benzo[*b*]thiophenes. Further studies, which are focused on the extension of the scope and the application of the reaction to the synthesis of bioactive products, are currently ongoing in our laboratory.

## Supporting Information

File 1 Full experimental details and copies of NMR spectral data.

## References

[1] Wu X-F, Neumann H, Beller M (2013). Chem Rev.

[2] Zeni G, Larock R C (2006). Chem Rev.

[3] Lipshutz B H (1986). Chem Rev.

[4] Lu H, Liu G-T (1992). Planta Med.

[5] Navarro E, Alonso S J, Trujillo J, Jorge E, Pérez C (2001). J Nat Prod.

[6] Cacchi S, Fabrizi G, Goggiamani A (2011). Org Biomol Chem.

[7] Flynn B L, Hamel E, Jung M K (2002). J Med Chem.

[8] Palkowitz A D, Glasebrook A L, Thrasher K J, Hauser K L, Short L L, Philips D L, Muehl B S, Sato M, Shetler P K, Cullinan G J (1997). J Med Chem.

[9] Wang X, Liu M, Xu L, Wang Q, Chen J, Ding J, Wu H (2013). J Org Chem.

[10] Kraus G A, Schroeder J D (2005). Synlett.

[11] Katritzky A R, Ji Y, Fang Y, Prakash I (2001). J Org Chem.

[12] Siddiqui I R, Waseem M A, Shamim S, Shireen, Srivastava A, Srivastava A (2013). Tetrahedron Lett.

[13] Eidamshaus C, Burch J D (2008). Org Lett.

[14] Liang Z, Hou W, Du Y, Zhang Y, Pan Y, Mao D, Zhao K (2009). Org Lett.

[15] Zeni G, Larock R C (2004). Chem Rev.

[16] Cho C-H, Neuenswander B, Lushington G H, Larock R C (2008). J Comb Chem.

[17] Cano R, Yus M, Ramón D J (2012). Tetrahedron.

[18] Liang Y, Tang S, Zhang X-D, Mao L-Q, Xie Y-X, Li J-H (2006). Org Lett.

[19] Arcadi A, Cacchi S, Di Giuseppe S, Fabrizi G, Marinelli F (2002). Org Lett.

[20] Okitsu T, Nakazawa D, Taniguchi R, Wada A (2008). Org Lett.

[21] Yue D, Yao T, Larock R C (2005). J Org Chem.

[22] Arcadi A, Cacchi S, Fabrizi G, Marinelli F, Moro L (1999). Synlett.

[23] Colobert F, Castanet A-S, Abillard O (2005). Eur J Org Chem.

[24] Ackermann L, Kaspar L T (2007). J Org Chem.

[25] Sun L-L, Deng C-L, Tang R-Y, Zhang X-G (2011). J Org Chem.

[26] Ma D, Xie S, Xue P, Zhang X, Dong J, Jiang Y (2009). Angew Chem, Int Ed.

[27] Kuhn M, Falk F C, Paradies J (2011). Org Lett.

[28] Guilarte V, Fernández-Rodríguez M A, García-García P, Hernando E, Sanz R (2011). Org Lett.

[29] Prasad D J C, Sekar G (2013). Org Biomol Chem.

[30] Tsuji H, Cantagrel G, Ueda Y, Chen T, Wan L-J, Nakamura E (2013). Chem – Asian J.

[31] Amii H, Uneyama K (2009). Chem Rev.

[32] Grecian S A, Hadida S, Warren S D (2005). Tetrahedron Lett.

[33] He C-Y, Fan S, Zhang X (2010). J Am Chem Soc.

[34] Nie J, Guo H-C, Cahard D, Ma J-A (2011). Chem Rev.

[35] O’Hagan D (2008). Chem Soc Rev.

[36] Lectard S, Hamashima Y, Sodeoka M (2010). Adv Synth Catal.

[37] Cho E J, Senecal T D, Kinzel T, Zhang Y, Watson D A, Buchwald S L (2010). Science.

[38] Matsuda S, Takahashi M, Monguchi D, Mori A (2009). Synlett.

[39] Jacubert M, Provot O, Peyrat J-F, Hamze A, Brion J-D, Alami M (2010). Tetrahedron.

[40] Pan W-B, Chen C-C, Wei L-L, Wei L-M, Wu M-J (2013). Tetrahedron Lett.

