# Supplementary material for: Copper-promoted hydration and annulation of 2-fluorophenylacetylene derivatives: from alkynes to benzo[b]furans and benzo[b]thiophenes
Source: Beilstein J Org Chem. 2014 Dec 4;10:2886–91. doi: 10.3762/bjoc.10.305 (PMC4273265; doi:10.3762/bjoc.10.305)
Supplement: File 1 — Full experimental details and copies of NMR spectral data. [file Beilstein_J_Org_Chem-10-2886-s001.pdf]

Supporting Information
for
Copper-promoted hydration and annulation of 2-fluorophenylacetylene derivatives: from alkynes to benzo[*b*]furans and benzo[*b*]thiophenes

Yibiao Li*[1], Liang Cheng[1], Xiaohang Liu[2], Bin Li[1] and Ning Sun[1]

Address: [1]School of Chemical & Environmental Engineering, Wuyi University, Jiangmen, Guangdong Province, 529090, China and [2]BASF Catalyst, 23800 Mercantile Road, Beachwood, Ohio 44124, USA

Email: Yibiao Li - leeyib268@126.com

* Corresponding author

Full experimental details and copies of NMR spectral data

List of Contents

A. General methods... S2

B Typical procedure for the copper-promoted reaction of 2-fluorophenylacetylene derivatives synthesis of benzo[*b*]furans.............. S2

C Typical procedure for the copper-promoted reaction of 2-fluorophenylacetylene derivatives synthesis of benzo[*b*]thiophenes S9

D ^{1}H NMR and ^{13}C NMR spectra for products.. S12

A. General methods

Unless otherwise noted, all commercial materials and solvents were used without further purification and all the reactions were carried out in a Schlenk tube equipped with a magnetic stir bar. ^{1}H NMR spectra were recorded in $CDCl_3$ at 400 MHz and ^{13}C NMR spectra were recorded in $CDCl_3$ at 100 MHz, respectively, ^{1}H and ^{13}C NMR were referenced to $CDCl_3$ at δ 7.26 and 77.0, respectively. GC–MS was obtained using electron ionization. HRMS was carried out on a MAT 95XP (Thermo). IR spectra were performed using potassium bromide pellets or liquid films between two potassium bromide pellets and a Brucker Vector 22 spectrometer. TLC was performed using commercially prepared 100–400 mesh silica gel plates (GF_{254}), and visualization was effected at 254 nm. All the other chemicals were purchased from Aldrich Chemicals. Commercial reagents were used without further purification.

B Typical procedure for the copper-promoted reaction of 2-fluorophenylacetylene derivatives synthesis of benzo[*b*]furans

A mixture of 1-fluoro-2-(2-phenylethynyl)benzene (1 mmol), CuI (20 mg, 0.1 mmol), KOH (112 mg, 2 mmol), KI (33 mg, 0.2 mmol) and DMSO (3 mL), was added successively in a 20 mL Schlenk tube. After stirring for 4 h at 80 °C, the solution was filtered though a small amount of silica gel. Then the residue was concentrated in vacuo and the crude was purified by flash chromatography with *n*-hexane/ethyl acetate (20/1, v/v) to afford the 2-phenylbenzofuran as a pale-yellow solid. All spectral data correspond to those given in the literature.

*2-Phenylbenzofuran (**2a**)* [1]

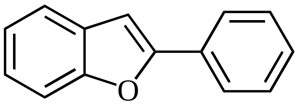

^{1}H NMR ($CDCl_3$, 400 MHz) δ 7.86 (d, J = 8.0 Hz, 2H), 7.57 (d, J = 7.6 Hz, 1H), 7.52 (d, J = 8.0 Hz, 1H), 7.43 (t, J = 7.8 Hz, 2H), 7.33 (t, J = 7.6 Hz, 1H), 7.29-7.20 (m, 2H), 7.00 (s, 1H); ^{13}C NMR ($CDCl_3$,100 MHz) δ 155.9, 154.9, 130.5, 129.2, 128.8 (2C), 128.5, 124.9 (2C), 124.2, 122.9, 120.9, 111.2, 101.3; MS (EI, 70 eV) m/z (%): 194, 165, 139, 97, 82.

*2-p-Tolylbenzofuran (**2b**)* [1]

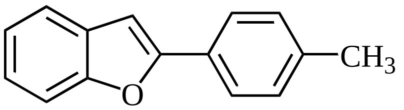

^{1}H NMR ($CDCl_3$, 400 MHz) δ 7.63 (d, J = 8.0 Hz, 2H), 7.56 (dd, J = 7.2 Hz, 16.8, 2H), 7.17−7.10 (m, 4H), 6.82 (s, 1H), 2.26 (s, 3H); ^{13}C NMR ($CDCl_3$, 100 MHz) δ 156.2, 154.8, 138.5, 129.4 (2C), 129.3, 127.7, 124.9 (2C), 124.0, 122.8, 120.7, 111.1, 100.5, 21.3; MS (EI, 70 eV) m/z (%): 208, 178, 165, 152, 104, 89.

*2-(2,4-Dimethylphenyl)benzofuran (**2c**)* [1]

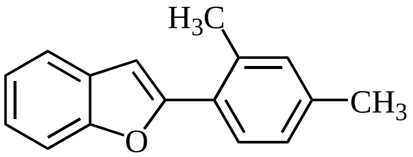

^{1}H NMR ($CDCl_3$, 400 MHz) δ 7.73 (d, J = 7.6 Hz, 1H), 7.55 (d, J = 7.2 Hz, 1H), 7.49 (d, J = 7.6 Hz, 1H), 7.17−7.18 (m, 2H), 7.12−7.06 (m, 2H), 6.80 (s, 1H), 2.51 (s, 3H), 2.32 (s, 3H); ^{13}C NMR ($CDCl_3$, 100 MHz) δ 155.6, 154.2, 138.4, 135.6, 132.0, 129.3, 128. 0, 127.1, 126.8, 124.0, 122.7, 120.7, 111.0, 104.4, 21.8, 21.1; MS (EI, 70 eV) m/z (%): 222, 207, 189, 178, 165, 152, 111; HRMS EI (m/z): calcd for $C_{16}H_{14}O$, 222.1045; found, 222.1041.

*2-(4-Methoxyphenyl)benzofuran (**2d**)* [1]

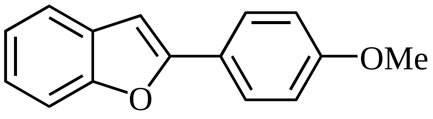

^{1}H NMR ($CDCl_3$, 400 MHz) δ 7.80 (d, J = 8.0 Hz, 2H), 7.56 (d, J = 7.6 Hz, 1H), 7.50 (d, J = 7.6 Hz, 1H), 7.28−7.20 (m, 2H), 6.99 (d, J = 8.0 Hz, 2H), 6.89 (s, 1H) 3.87 (s, 3H); ^{13}C NMR ($CDCl_3$, 100 MHz) δ 160.0,

156.0, 154.7, 129.5, 126.4 (2C), 123.7, 123.3, 122.8, 120.5, 114.2 (2C), 111.0, 99.7, 55.4; MS (EI, 70 eV) m/z (%): 224, 209, 181, 152, 112.

*2-(Biphenyl-4-yl)benzofuran (**2e**)* [1]

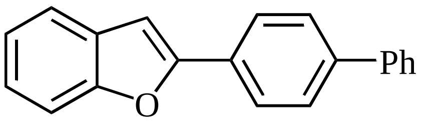

^{1}H NMR ($CDCl_3$, 400 MHz) *δ* 7.95 (d, *J* = 7.2 Hz, 2H), 7.68 (dd, *J* = 16.2, 7.2 Hz, 4H), 7.58 (dd, *J* = 21.8, 7.2 Hz, 2H), 7.48 (t, *J* =7.2 Hz, 2H), 7.39 (d, *J* = 6.2 Hz, 1H), 7.31-7.26 (m, 2H), 7.07 (s, 1H); ^{13}C NMR ($CDCl_3$, 100 MHz) *δ* 155.7, 155.0, 141.2, 140.4, 129.4, 129.3, 128.9 (2C), 127.6, 127.4 (2C), 127.0 (2C), 125.3 (2C), 124.3, 123.0, 120.9, 111.2, 101.4; MS (EI, 70 eV) m/z (%): 270, 239, 165, 135, 96, 73.

*2-(3-Chlorophenyl)benzofuran (**2f**)* [1]

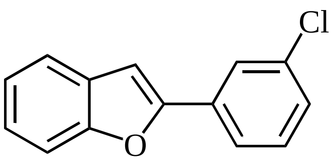

^{1}H NMR ($CDCl_3$, 400 MHz) *δ* 7.81 (s, 1H), 7.66 (d, *J* = 7.2 Hz, 1H), 7.54 (d, *J* = 7.6 Hz, 1H), 7.48 (d, *J* = 8.0 Hz, 1H), 7.32-7.19 (m, 4H), 6.96 (s, 1H); ^{13}C NMR ($CDCl_3$, 100 MHz) *δ* 154.9, 154.2, 134.8, 132.1, 130.0, 128.9, 128.3, 124.8, 124.7, 123.1, 122.9, 121.1, 111.2, 102.3; MS (EI, 70 eV) m/z (%): 228, 199, 165, 139, 114, 82.

*2-(4-Chlorophenyl)benzofuran (**2g**)* [1]

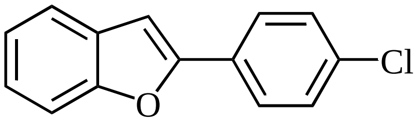

^{1}H NMR ($CDCl_3$, 400 MHz) *δ* 7.72 (d, *J* = 8.8 Hz, 2H), 7.53 (d, *J* = 7.2 Hz, 1H), 7.48 (d, *J* = 8.0 Hz, 1H), 7.36 (d, J = 8.8 Hz, 2H), 7.29−7.19 (m, 2H), 6.92 (s, 1H); ^{13}C NMR ($CDCl_3$, 100 MHz) *δ* 154.9, 154.7, 134.2, 129.0, 129.0 (2C), 128.9, 126.1 (2C), 124.5, 123.1, 121.0, 111.2, 101.7; MS (EI, 70 eV) m/z (%): 228, 199, 165, 139, 114.

*2-(3-Fluorophenyl)benzofuran (**2h**)* [2]

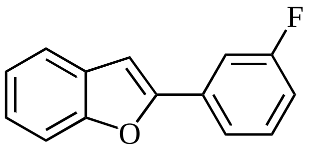

^{1}H NMR (400 MHz, $CDCl_3$) δ 7.60-7.49 (m, 4H), 7.34 (dd, J = 14.3, 7.5 Hz, 1H), 7.24 (dt, J = 25.0, 7.2 Hz, 2H), 7.00 (t, J = 8.4 Hz, 1H), 6.99 (s, 1H); ^{13}C NMR ($CDCl_3$, 100 MHz) δ 163.1 (d, $J_{C\text{-}F}$ = 244.2 Hz), 154.9, 154.4 (d, $J_{C\text{-}F}$ = 3.0 Hz), 132.5 (d, $J_{C\text{-}F}$ = 8.4 Hz), 130.3 (d, $J_{C\text{-}F}$ = 8.4 Hz), 128.9, 124.7, 123.1, 121.1, 120.5 (d, $J_{C\text{-}F}$ = 2.9 Hz), 115.3 (d, J = 21.2 Hz), 111.9, 111.5 (d, $J_{C\text{-}F}$ = 23.5 Hz), 111.2, 102.3; MS (EI, 70 eV) m/z (%): 212, 183, 157, 106, 91.

*2-(4-Fluorophenyl)benzofuran (**2i**)* [3]

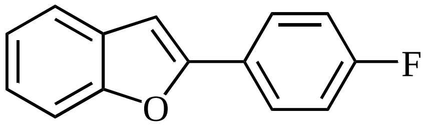

^{1}H NMR ($CDCl_3$, 400 MHz) δ 7.84−7.81 (m, 2H), 7.57 (d, J = 7.6 Hz, 1H), 7.50 (d, J = 8.1 Hz, 1H), 7.27 (t, J = 7.5 Hz, 1H), 7.22 (t, J = 7.8 Hz, 1H), 7.13 (t, J = 8.7 Hz, 2H), 6.94 (s, 1H); ^{13}C NMR ($CDCl_3$, 100 MHz) δ 162.9 (d, $J_{C\text{-}F}$ = 247.3 Hz), 155.1, 154.9, 129.2, 126.8 (d, $J_{C\text{-}F}$ = 3.8 Hz, 2C), 126.7, 124.3, 123.0, 120.9, 115.9 (d, $J_{C\text{-}F}$ = 22.9 Hz, 2C), 111.2, 101.0 (d, $J_{C\text{-}F}$ = 1.5 Hz); MS (EI, 70 eV) m/z (%): 212, 183, 157, 106, 91, 77.

*2-(Thiophen-2-yl)benzofuran (**2j**)* [4]

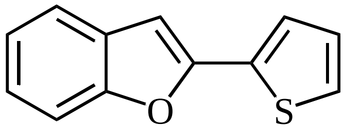

^{1}H NMR (400 MHz, $CDCl_3$) δ 7.51-7.44 (m, 3H), 7.23 (ddd, J = 19.6, 12.5, 6.1 Hz, 3H), 7.05 (t, J = 4.2 Hz, 1H), 6.81 (s, 1H); ^{13}C NMR (100 MHz, $CDCl_3$) δ 154.5, 151.2, 133.2, 129.1, 127.8, 125.7, 124.6, 124.2, 123.1, 120.7, 111.0, 101.1; MS (EI, 70 eV) m/z (%): 200, 171, 155, 145, 127, 100.

*6-Methyl-2-phenylbenzofuran (**2k**)* [5]

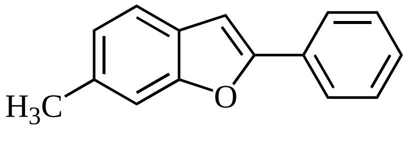

^{1}H NMR ($CDCl_3$, 400 MHz) δ 7.77 (d, J = 8.0 Hz, 2H), 7.35 (t, J = 6.0 Hz, 3H), 7.26-7.23 (m, 2H), 6.97 (d, J = 7.6 Hz, 1H), 6.89 (s, 1H), 2.4

(s, 3H); ^{13}C NMR ($CDCl_3$, 100 MHz) δ 155.3, 155.3, 134.5, 130.7, 128.7 (2C), 128.3, 126.7, 124.7 (2C), 124.3, 120.3, 111.4, 101.2, 21.7; MS (EI, 70 eV) m/z (%): 208, 178, 165, 152, 104, 77.

*6-Chloro-2-phenylbenzofuran (**2l**)* [6]

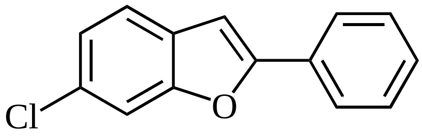

^{1}H NMR ($CDCl_3$, 400 MHz) δ 7.83 (d, J = 7.6 Hz, 2H), 7.53 (s, 1H), 7.49-7.43 (m, 2H), 7.38 (t, J = 8.8 Hz, 1H), 7.27-7.20 (m, 2H), 6.98 (s, 1H); ^{13}C NMR ($CDCl_3$, 100 MHz) δ 156.7, 154.9, 129.9, 128.8 (3C), 128.7, 127.9, 124.9 (2C), 123.7, 121.3, 111.7, 101.0; MS (EI, 70 eV) m/z (%): 228, 199, 165, 139, 114, 77.

*6-Bromo-2-phenylbenzofuran (**2m**)*

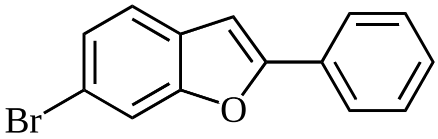

IR (KBr): v_{max} = 3091, 1448, 1019, 919, 868, 822, 762 cm^{-1}; ^{1}H NMR ($CDCl_3$, 400 MHz) δ 7.82 (d, J = 7.2 Hz, 2H), 7.68 (s, 1H), 7.46-7.40 (m, 3H), 7.38-7.33 (m, 2H), 6.95 (s, 1H); ^{13}C NMR ($CDCl_3$, 100 MHz) δ 156.6, 155.1, 129.9, 128.8 (3C), 126.3, 124.9 (2C), 121.7, 117.3, 114.6, 101.0; MS (EI, 70 eV) m/z (%): 274, 272, 193, 165, 139, 115, 82; HRMS EI (m/z): calcd for $C_{14}H_9BrO$, 271.9837; found, 271.9833.

*6-Fluoro-2-phenylbenzofuran (**2n**)*

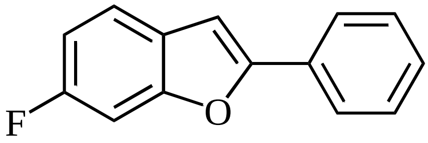

IR (KBr): v_{max} = 3104, 3084, 2924, 1601, 1450, 1132, 853, 821, 761 cm^{-1}; ^{1}H NMR ($CDCl_3$, 400 MHz) δ 7.81 (d, J = 7.6 Hz, 2H), 7.48-7.41 (m, 3H), 7.35-7.32 (m, 1H), 7.23 (d, J = 8.4 Hz, 1H), 6.99 (t, J = 9.2 Hz, 1H), 6.95 (s, 1H); ^{13}C NMR ($CDCl_3$, 100 MHz) δ 160.8 (d, J = 240.7 Hz), 156.7 (d, J = 4.3 Hz), 154.8 (d, J = 13.6 Hz), 130.2, 128.8 (2C), 128.6, 124.7 (2C), 123.7, 121.1 (d, J = 9.9 Hz), 111.2 (d, J = 23.8 Hz), 100.9, 99.0 (d, J = 26.6 Hz); MS

(EI, 70 eV) m/z (%): 212, 183, 157, 106, 91; HRMS EI (m/z): calcd for $C_{14}H_9FO$, 212.0637; found, 212.0631.

*6-Fluoro-2-(2,4-dimethylphenyl)benzofuran (**2o**)*

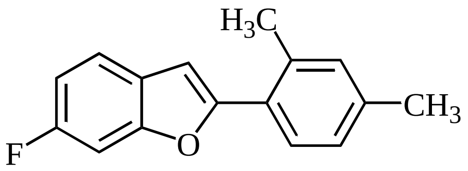

IR (KBr): ν_{max} = 3037, 2959, 2924, 1599, 1486, 1267, 838, 810 cm^{-1}; ^1H NMR ($CDCl_3$, 400 MHz) δ 7.69 (d, J = 8.8 Hz, 1H), 7.47 (dd, J = 8.8, 5.4 Hz, 1H), 7.25-7.17 (m, 1H), 7.10 (s, 1H), 7.09 (s, 1H), 7.03-6.93 (m, 1H), 6.78 (s, 1H), 2.51 (s, 3H), 2.35 (s, 3H); ^{13}C NMR ($CDCl_3$, 100 MHz) δ 160.7 (d, J = 240.3 Hz), 156.7 (d, J = 4.3 Hz), 154.2 (d, J = 13.5 Hz), 138.5, 135.4, 132.0, 127.9, 126.9 (2C), 125.5, 120.9 (d, J = 9.9 Hz), 111.2 (d, J = 23.9 Hz), 104.1, 98.7 (d, J = 36.4 Hz), 21.8, 21.1; MS (EI, 70 eV) m/z (%): 240, 225, 196, 120, 98; HRMS EI (m/z): calcd for $C_{16}H_{13}FO$, 240.0950; found, 240.0945.

*2-(4-Dimethylaminophenyl)-6-fluorobenzofuran (**2p**)*

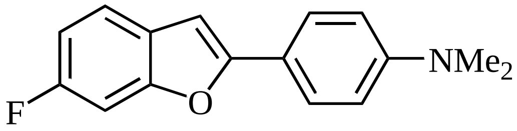

IR (KBr): ν_{max} = 3105, 3036, 2921, 1614, 1495, 1453, 1256, 1170, 1019, 798, 749 cm^{-1}; ^1H NMR ($CDCl_3$, 400 MHz) δ 7.61 (d, J = 8.0 Hz, 2H), 7.32 (t, J = 6.8 Hz, 1H), 7.12 (d, J = 8.8 Hz, 1H), 6.87 (t, J = 9.2 Hz, 1H), 6.69 (s, 1H), 6.67 (s, 2H), 2.93 (s, 6H); ^{13}C NMR ($CDCl_3$, 100 MHz) δ 160.2 (d, J = 238.9 Hz), 157.8 (d, J = 3.7 Hz), 154.4 (d, J = 3.5 Hz), 150.5, 126.0 (d, J = 1.2 Hz), 125.9 (4C), 120.1 (d, J = 9.8 Hz,), 112.2, 110.7 (d, J = 23.7 Hz), 98.7 (d, J = 25.5 Hz), 97.7, 40.3 (2C); MS (EI, 70 eV) m/z (%): 255, 212, 239, 183, 127; HRMS EI (m/z): calcd for $C_{16}H_{14}FNO$, 255.1059; found, 255.1053.

*6,7-Difluoro-2-(4-fluorophenyl)benzofuran (**2q**)*

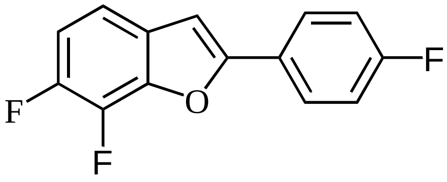

IR (KBr): v_{max} = 3116, 3056, 2921, 2849, 1609, 1499, 1306, 1229, 1036, 838, 815 cm^{-1}; ^{1}H NMR ($CDCl_3$, 400 MHz) δ 7.82 (dd, *J* = 8.6, 5.4 Hz, 2H), 7.22 (dd, *J* = 8.6, 4.4 Hz, 1H), 7.15 (t, *J* = 8.6 Hz, 2H), 7.10-7.03 (m, 1H), 6.91 (d, *J* = 2.8 Hz, 1H); ^{13}C NMR ($CDCl_3$, 100 MHz) δ 163.1 (d, *J* = 248.1 Hz), 157.0 (d, *J* = 4.1 Hz), 148.2 (dd, *J* = 9.4 Hz, 241.2 Hz), 142.4 (dd, *J* = 4.8, 8.0 Hz), 140.0 (d, *J* = 16.7, 251.1 Hz), 127.9, 126.8 (d, *J* = 8.2 Hz, 2C), 125.9 (d, *J* = 3.4 Hz), 116.0 (d, *J* = 220.0 Hz, 2C), 115.1 (dd, *J* = 8.1, 4.6 Hz), 112.6 (d, *J* = 20.1 Hz), 100.8 (d, *J* = 1.8 Hz)；MS (EI, 70 eV) m/z (%): 248, 219, 201, 124; HRMS EI (m/z): calcd for $C_{14}H_7F_3O$, 248.0449; found, 248.0442.

*2-(3-(Benzofuran-2-yl)phenyl)benzofuran (**2r**)*

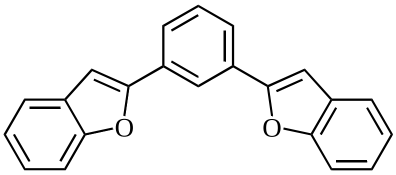

IR (KBr): v_{max} = 3031, 2922, 2851, 1704, 1450, 1367, 1257, 1212, 747 cm^{-1}; ^{1}H NMR (CDCl3, 400 MHz) δ 8.38 (s, 1H), 7.84 (d, *J* = 7.6 Hz, 2H), 7.63-7.52 (m, 6H), 7.34-7.28 (m, 3H), 7.2 (s, 2H); ^{13}C NMR (CDCl3, 100 MHz) δ 155.4, 155.0, 131.1, 129.3, 129.1, 124.9, 124.5, 123.0, 121.2, 121.0, 111.3, 102.0; MS (EI, 70 eV) m/z (%): 310, 252, 191, 155, 126, 96, 73; HRMS EI (m/z): calcd for $C_{22}H_{14}O_2$, 310.0994; found, 310.0988.

*2-(Benzofuran-2-yl)benzofuran (**2s**)* [7]

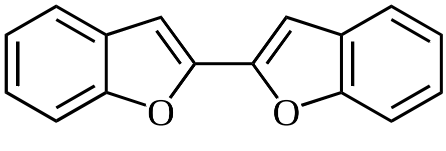

^{1}H NMR (400 MHz, $CDCl_3$) δ = 7.63 (d, *J* = 7.2 Hz, 2H), 7.54 (d, *J* = 8.0 Hz, 2H), 7.34 (t, *J* = 7.6 Hz, 2H), 7.29-7.26 (m, 2H), 7.17 (s, 2H); ^{13}C NMR (100 MHz, CDCl3) δ = 155.1, 147.7, 128.5, 125.1, 123.3, 121.4, 111.3, 103.7; MS (EI, 70 eV) m/z (%): 234, 205, 176, 152, 117.

*2-(2-Bromophenyl)benzofuran (**2t**)* [8]

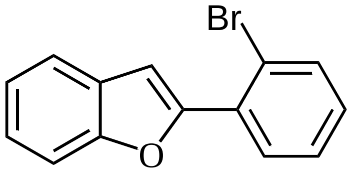

^{1}H NMR ($CDCl_3$, 400 MHz) *δ* 7.74-7.69 (m, 2H), 7.56 (d, *J* = 7.6 Hz, 1H), 7.50 (d, *J* = 8.0 Hz, 1H), 7.41 (t, *J* = 8.0 Hz, 1H), 7.29 (t, *J* = 7.2 Hz, 1H), 7.22 (t, *J* = 7.2 Hz, 1H), 7.16 (d, *J* = 8.0 Hz, 1H), 7.01 (s, 1H); ^{13}C NMR ($CDCl_3$, 100 MHz) *δ* 154.9, 154.1, 149.7, 132.4, 130.2, 128.9, 124.9, 123.2, 123.1, 121.2, 120.6, 117.3, 111.3, 102.6; MS (EI, 70 eV) m/z (%): 274, 272, 165, 137, 83.

*2-(2-Chlorophenyl)benzofuran (**2v**)* [9]

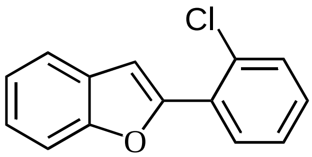

^{1}H NMR ($CDCl_3$, 400 MHz) *δ* 8.03 (d, *J* = 8.0 Hz, 1H), 7.61 (d, *J* = 7.6 Hz, 1H), 7.52-7.45 (m, 3H), 7.36−7.28 (m, 2H), 7.25-7.20 (m, 2H); ^{13}C NMR ($CDCl_3$, 100 MHz) *δ* 154.1, 151.9, 131.3, 130.8, 129.0, 129.0, 128.9, 128.9, 126.9, 124.9, 122.9, 121.4, 111.0, 107.3; MS (EI, 70 eV) m/z (%): 228, 199, 165, 139, 114, 82.

C Typical procedure for the copper-promoted reaction of 2-fluorophenylacetylene derivatives synthesis of benzo[*b*]thiophenes

A mixture of 2-fluorophenylacetylenes (1 mmol), CuI (20 mg, 0.1 mmol), $Na_2S{\cdot}9H_2O$ (2 mmol) and DMSO (3 mL), was added successively in a 20 mL Schlenk tube. After stirring for 8 h at 60 °C, the solution was filtered though a small amount of silica gel. Then the residue was concentrated in vacuo and the crude was purified by flash chromatography with *n*-hexane/ethyl acetate (20/1, v/v) to afford the benzothiophenes **3a**–**c** as a pale-yellow solid. All spectral data correspond to those given in the literature.

*2-phenylbenzo[b]thiophene (**3a**)*[10]

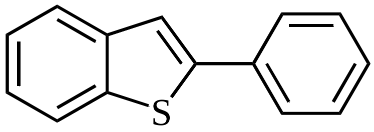

^{1}H NMR (400 MHz, $CDCl_3$) δ = 7.78 (dd, *J* = 23.0, 7.8 Hz, 2H), 7.70 (d, *J* = 7.8 Hz, 2H), 7.52 (s, 1H), 7.40 (t, *J* = 7.6 Hz, 2H), 7.31-7.35 (m, 3H); ^{13}C NMR (100 MHz, CDCl3) δ = 144.2, 140.7, 139.5, 134.3, 128.9 (2C), 128.2, 126.5 (2C), 124.5, 124.3, 123.5, 122.2, 119.4; MS (EI, 70 eV) m/z (%): 210, 202, 178, 165.

*2-(3-chlorophenyl)benzo[b]thiophene (**3b**)*[10]

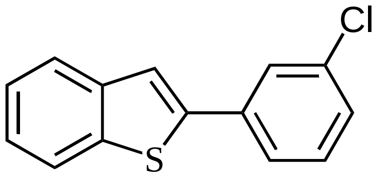

^{1}H NMR (400 MHz, $CDCl_3$) δ = 7.76 (dd, *J* = 22.4, 7.6 Hz, 2H), 7.67 (s, 1H), 7.53 (d, *J* = 6.8 Hz, 1H), 7.49 (s, 1H), 7.36-7.24 (m, 4H); ^{13}C NMR (100 MHz, CDCl3) δ = 142.4, 140.4, 139.5, 136.0, 134.8, 130.1, 128.1, 126.4, 124.7, 124.6, 124.6, 123.7, 122.3, 120.3; MS (EI, 70 eV) m/z (%): 246, 244.

*2-(4-chlorophenyl)benzo[b]thiophene (**3c**)*[10]

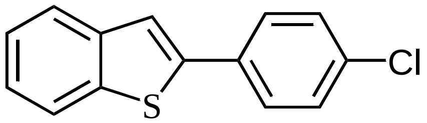

^{1}H NMR (400 MHz, $CDCl_3$) δ = 7.78 (dd, *J* = 22.5, 7.6 Hz, 2H), 7.61 (d, *J* = 8.2 Hz, 2H), 7.50 (s, 1H), 7.41-7.28 (m, 4H); ^{13}C NMR (100 MHz, CDCl3) δ = 142.8, 140.6, 139.5, 134.1, 132.8, 129.1 (2C), 127.6 (2C), 124.7, 124.6, 123.6, 122.3, 119.9; MS (EI, 70 eV) m/z (%):246, 244.

References

1. Wang, X.; Liu, M.; Xu, L.; Wang, Q.; Chen, J.; Ding, J.; Wu, H. *J. Org. Chem*. **2013**, *78*, 5273-5281.
2. Isono, N.; Lautens, M. *Org. Lett*. **2009**, *11*, 1329-1331.
3. Chittimalla, S. K.; Chang, T. C.; Liu, T. C.; Hsieh, H. P.; Liao, C. C. *Tetrahedron*. **2008,** *64*, 2586-2595.

4. Nakamura, M.; Ilies, L.; Otsubo, S.; Nakamura, E. *Angew. Chem. Int. Ed*. **2006**, *45*, 944-947.
5. Ishii, H.; Ishikawa, Y.; Mizukami, K.; Mitsui, H.; Ikeda, N. *Chem. Pharm. Bull.* **1971**, *19*, 970-97.
6. Cho, E. J.; Senecal, T.D.; KInzel, T.; Zhang, Y.; Watson, D.A.; Buchwald, S.L. *Science*. **2010**, *328*, 1679-1681.
7. Matsuda, S.; Takahashi, M.; Monguchi, D.; Mori, A. *Synlett*. **2009**, *12*, 1941-1944.
8. Csékei, M.; Nolták, Z.; Kotschy, A. *Org. Lett*. **2009**, *11*, 1329-1331.
9. Siddiqui, I. R.; Waseem, M. A.; Shamim, S.; Shireen, Srivastava, A.; Srivastava, A. *Tetrahedron Letters*. **2013**, *54*, 4154-4158.
10. (a) Sun, L. L.; Deng, C. L.; Tang, R. Y.; Zhang, X. G. *J. Org. Chem*. **2011**, *76*, 7546-7550; (b) Prasad, D. J. C.; Sekar, G. *Org. Biomol. Chem*. **2013**, *11*, 1659-1665; (c) Kuhn, M.; Falk, F. C.; Paradies, J. *Org. Lett*. **2011**, *13*, 4100-4103.

D ^{1}H NMR and ^{13}C NMR spectra for products

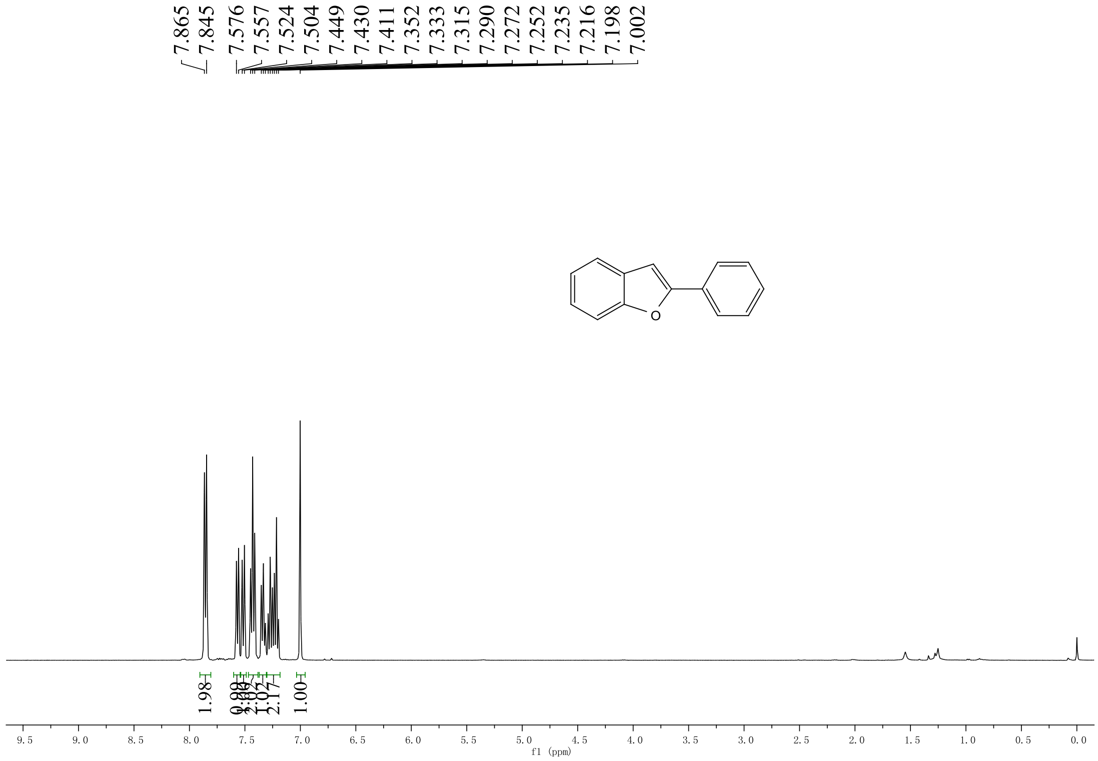

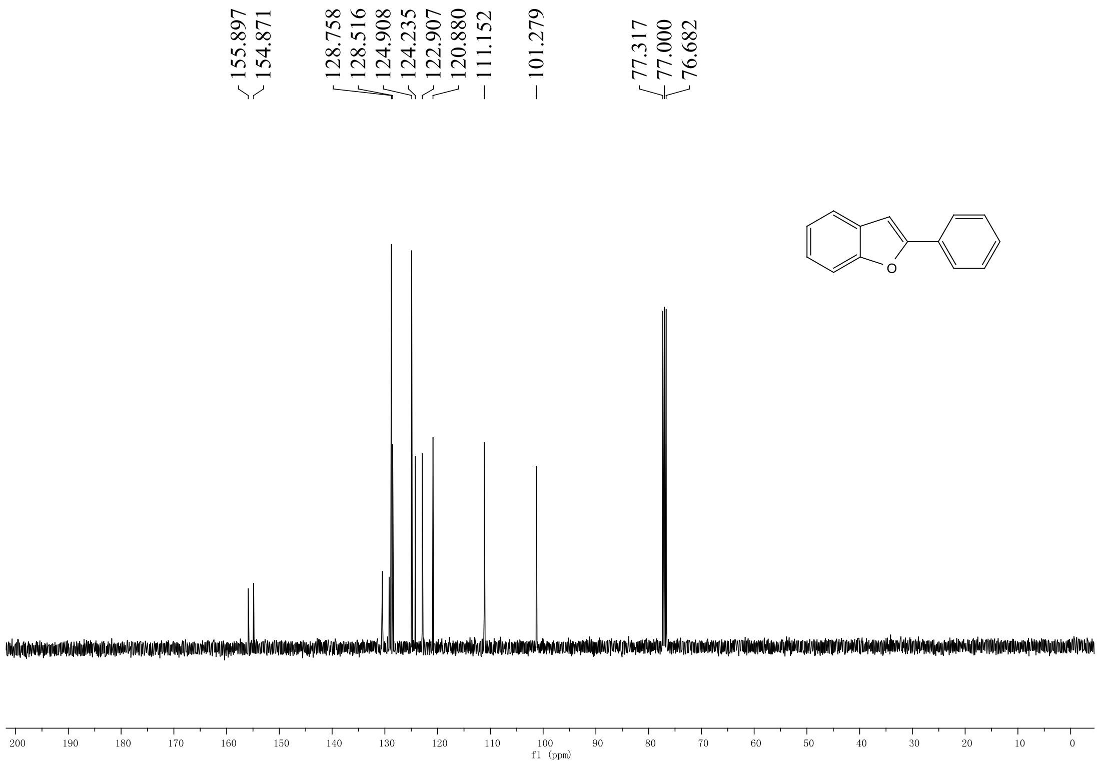

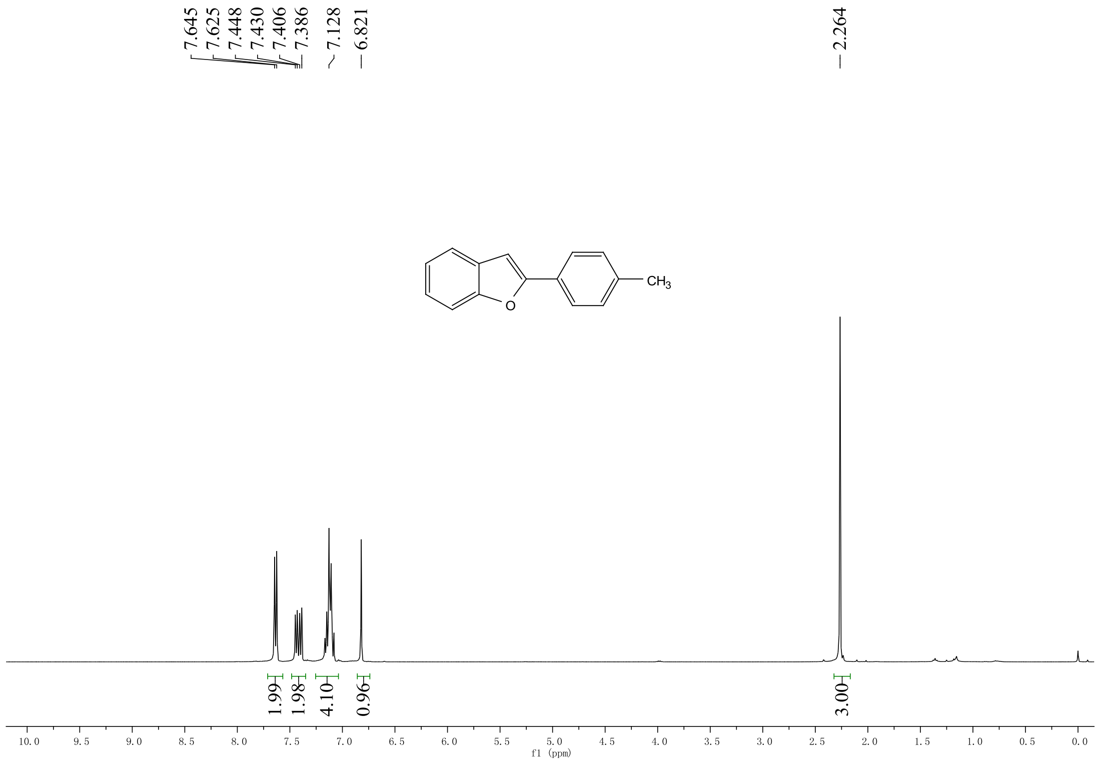
7.645
7.625
7.448
7.430
7.406
7.386
7.128
6.821
2.264
CH3
O
1.99
1.98
4.10
0.96
3.00
f1 (ppm)

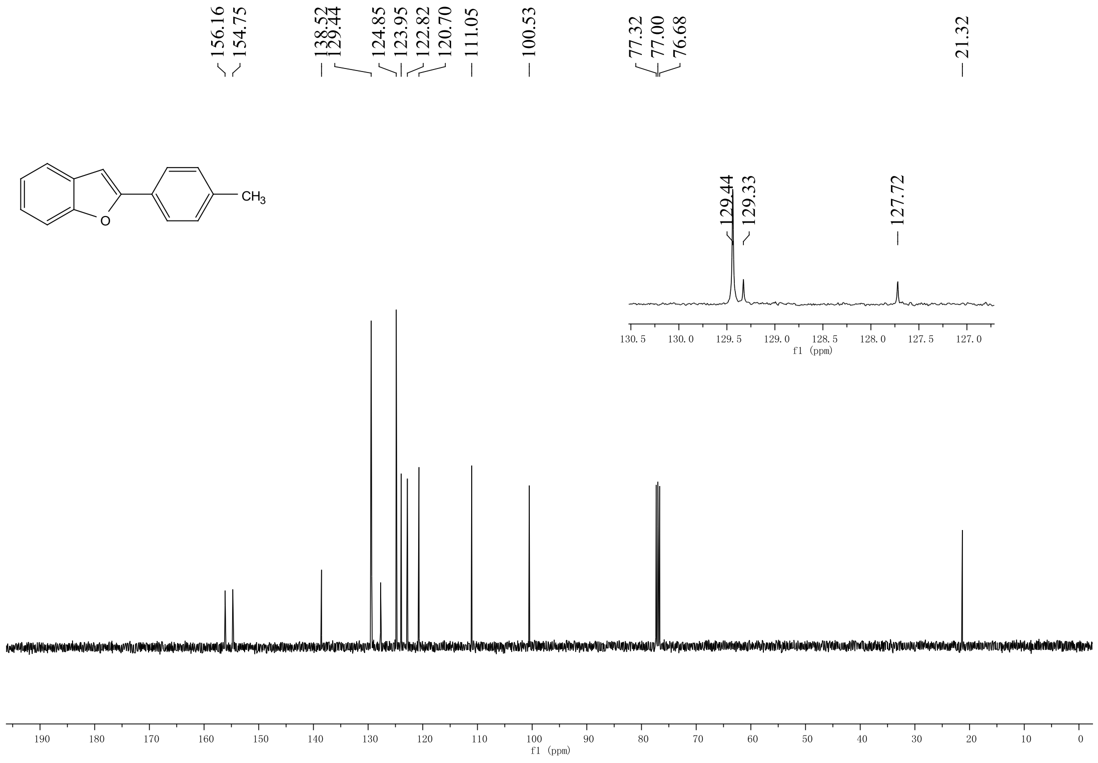
156.16
154.75
138.52
129.44
124.85
123.95
122.82
120.70
111.05
100.53
77.32
77.00
76.68
21.32
CH3
O
129.44
129.33
127.72
f1 (ppm)

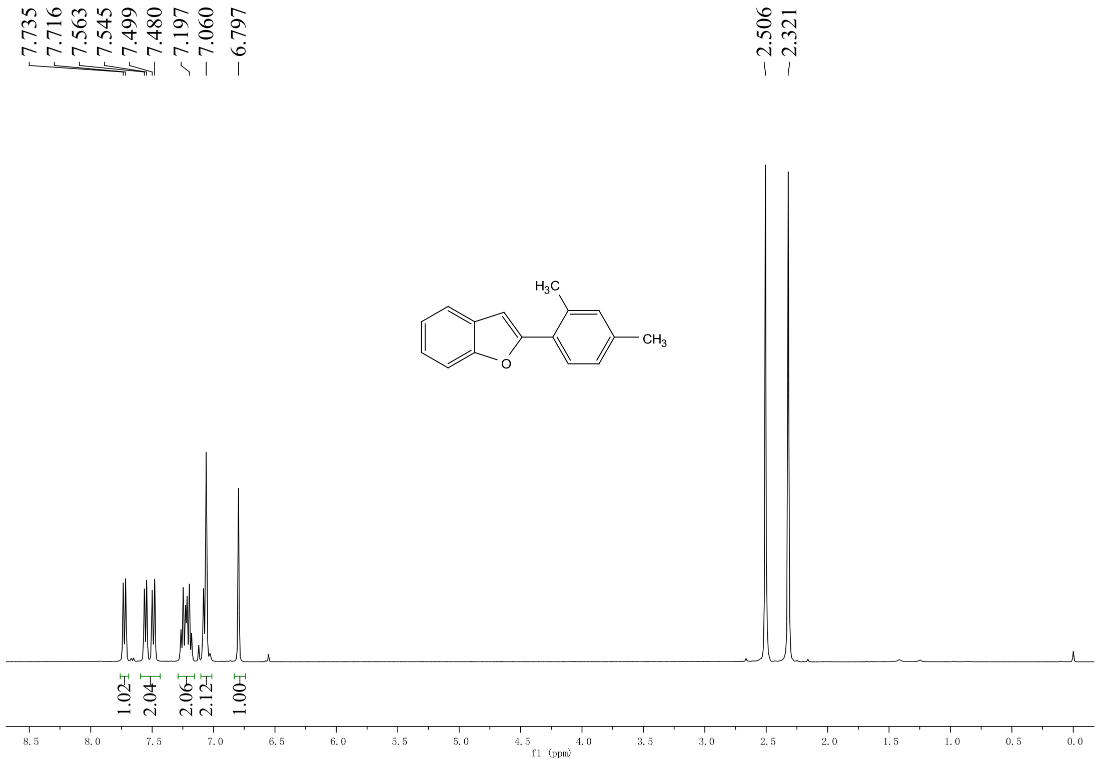
7.735
7.716
7.563
7.545
7.499
7.480
7.197
7.060
6.797
2.506
2.321
H3C
CH3
O
1.02
2.04
2.06
2.12
1.00
f1 (ppm)

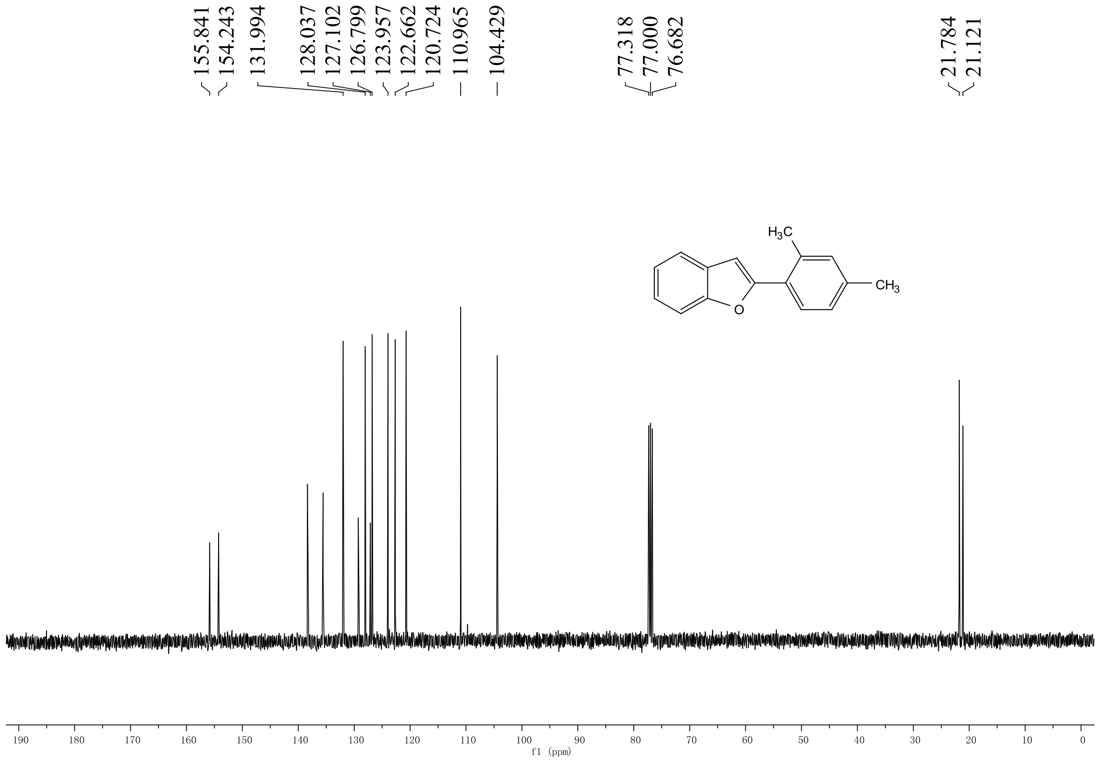
155.841
154.243
131.994
128.037
127.102
126.799
123.957
122.662
120.724
110.965
104.429
77.318
77.000
76.682
21.784
21.121
H3C
CH3
O
f1 (ppm)

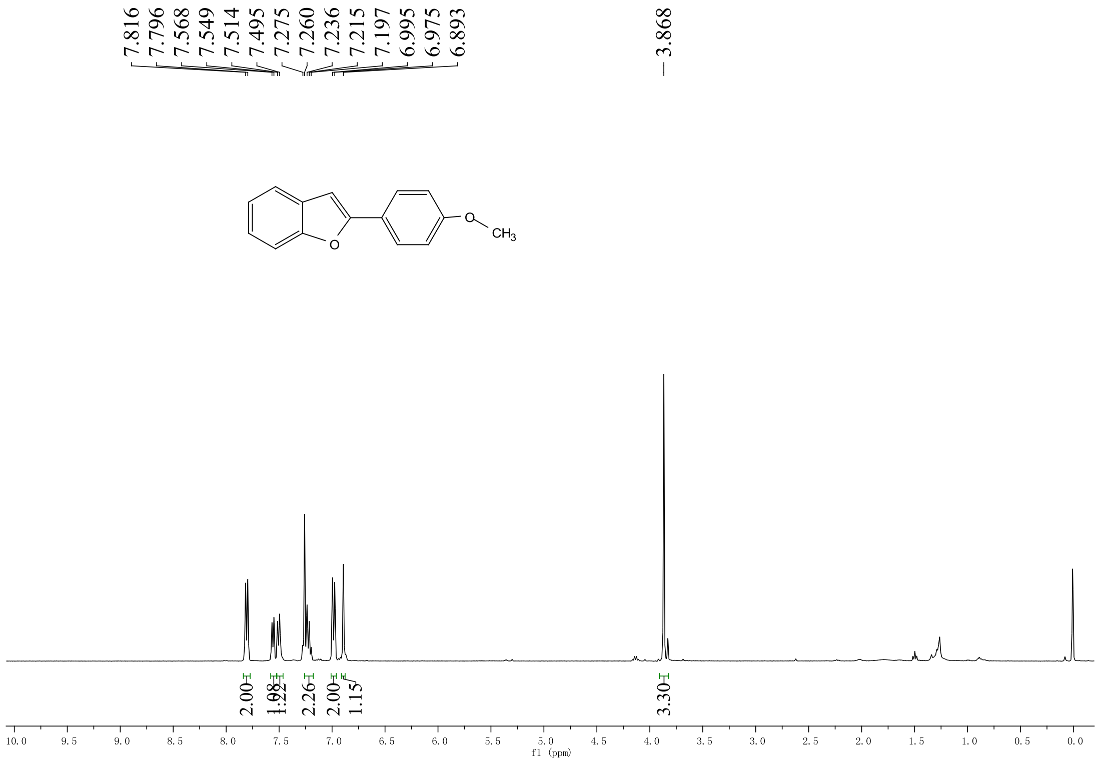
7.816
7.796
7.568
7.549
7.514
7.495
7.275
7.260
7.236
7.215
7.197
6.995
6.975
6.893
3.868
O
O
CH3
2.00
1.08
1.22
2.26
2.00
1.15
3.30
f1 (ppm)

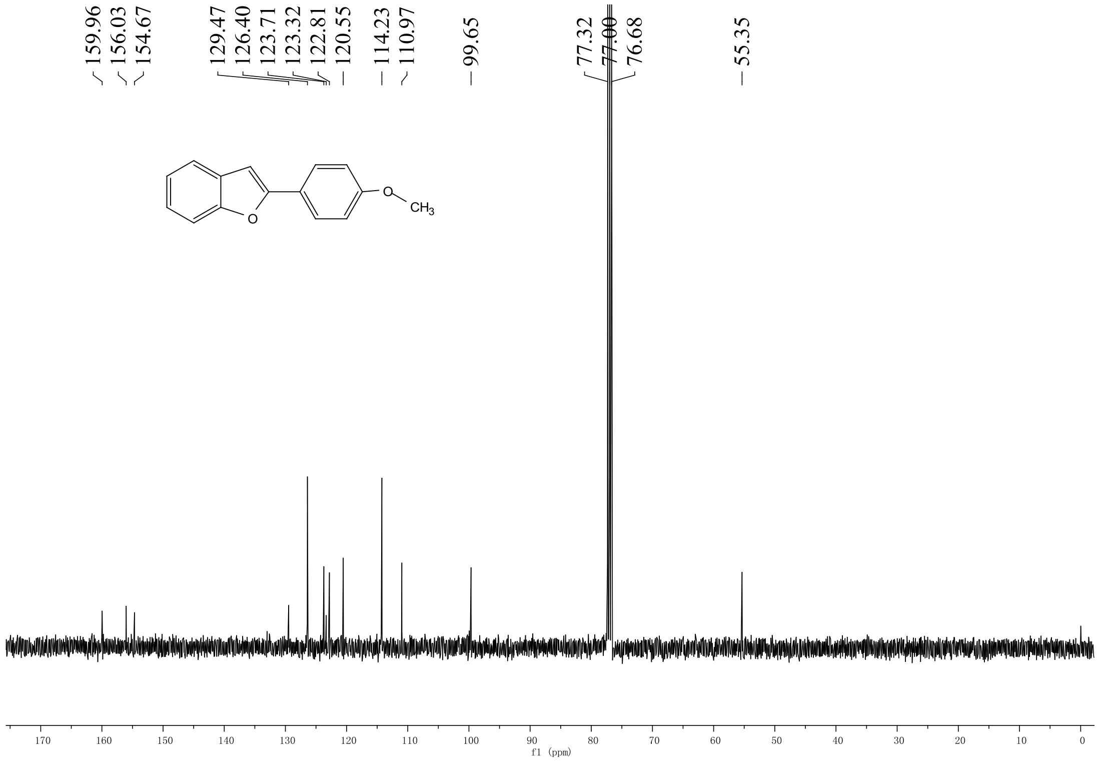
159.96
156.03
154.67
129.47
126.40
123.71
123.32
122.81
120.55
114.23
110.97
99.65
77.32
77.00
76.68
55.35
O
O
CH3
f1 (ppm)

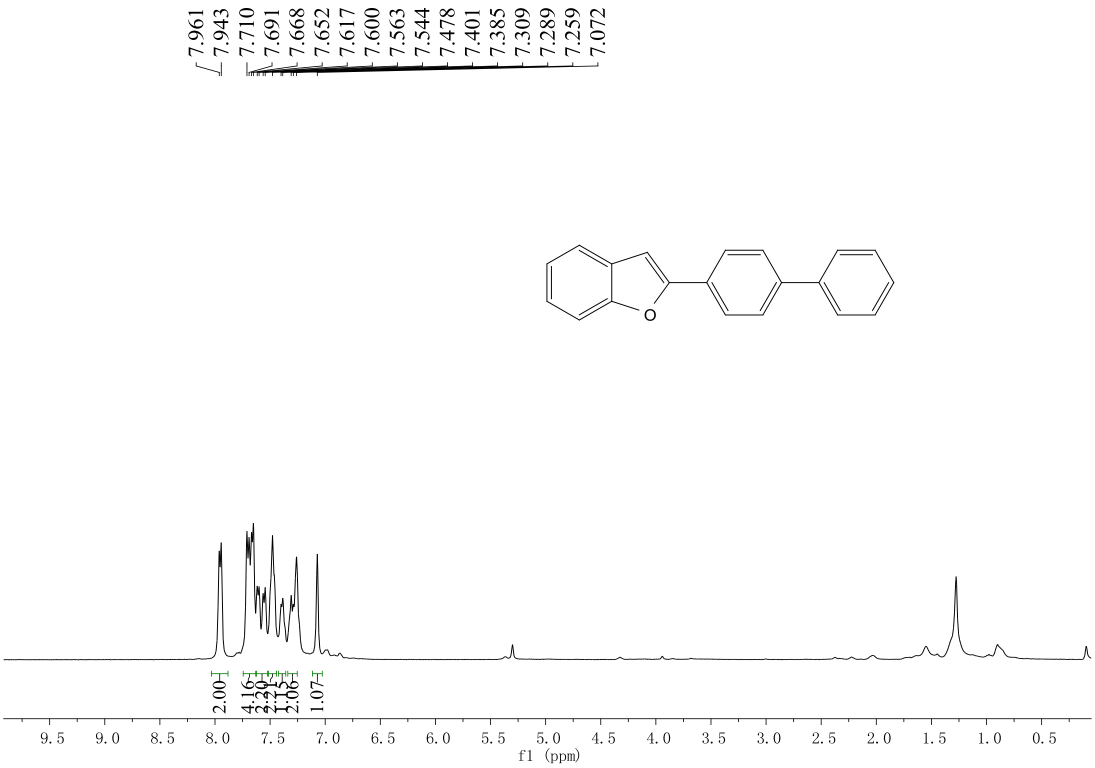
7.961
7.943
7.710
7.691
7.668
7.652
7.617
7.600
7.563
7.544
7.478
7.401
7.385
7.309
7.289
7.259
7.072
O
2.00
4.16
2.20
2.15
1.15
2.06
1.07
9.5 9.0 8.5 8.0 7.5 7.0 6.5 6.0 5.5 5.0 4.5 4.0 3.5 3.0 2.5 2.0 1.5 1.0 0.5
f1 (ppm)

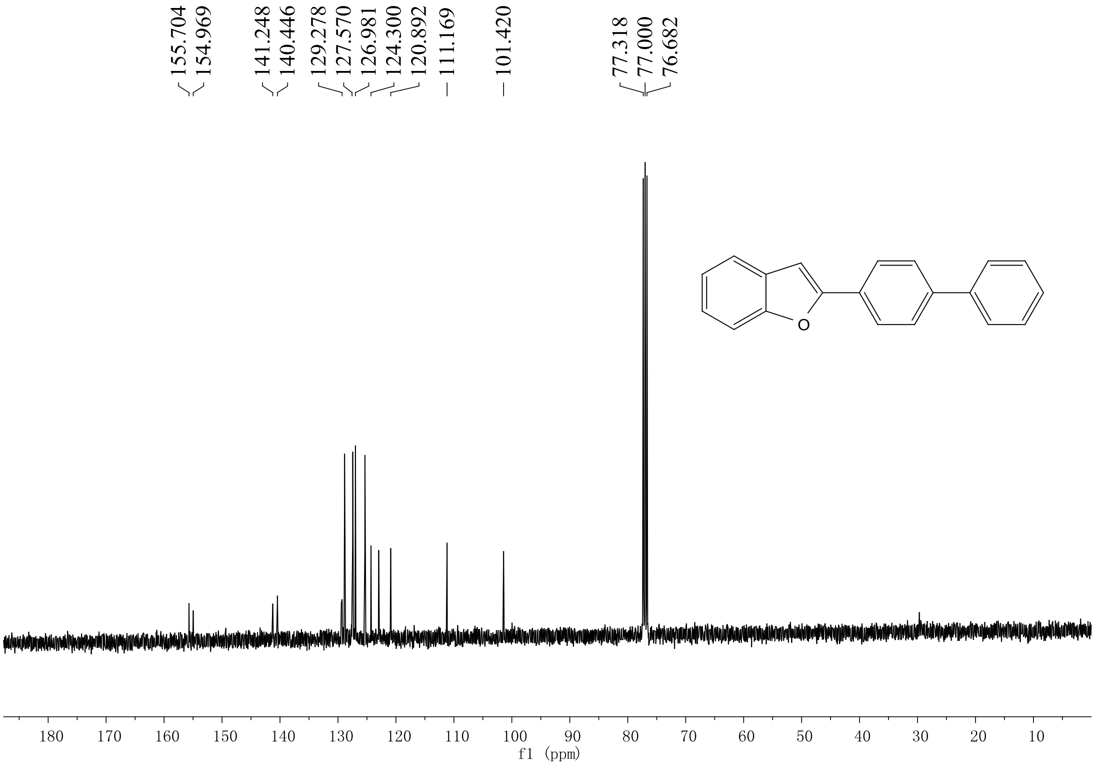
155.704
154.969
141.248
140.446
129.278
127.570
126.981
124.300
120.892
111.169
101.420
77.318
77.000
76.682
O
180 170 160 150 140 130 120 110 100 90 80 70 60 50 40 30 20 10
f1 (ppm)

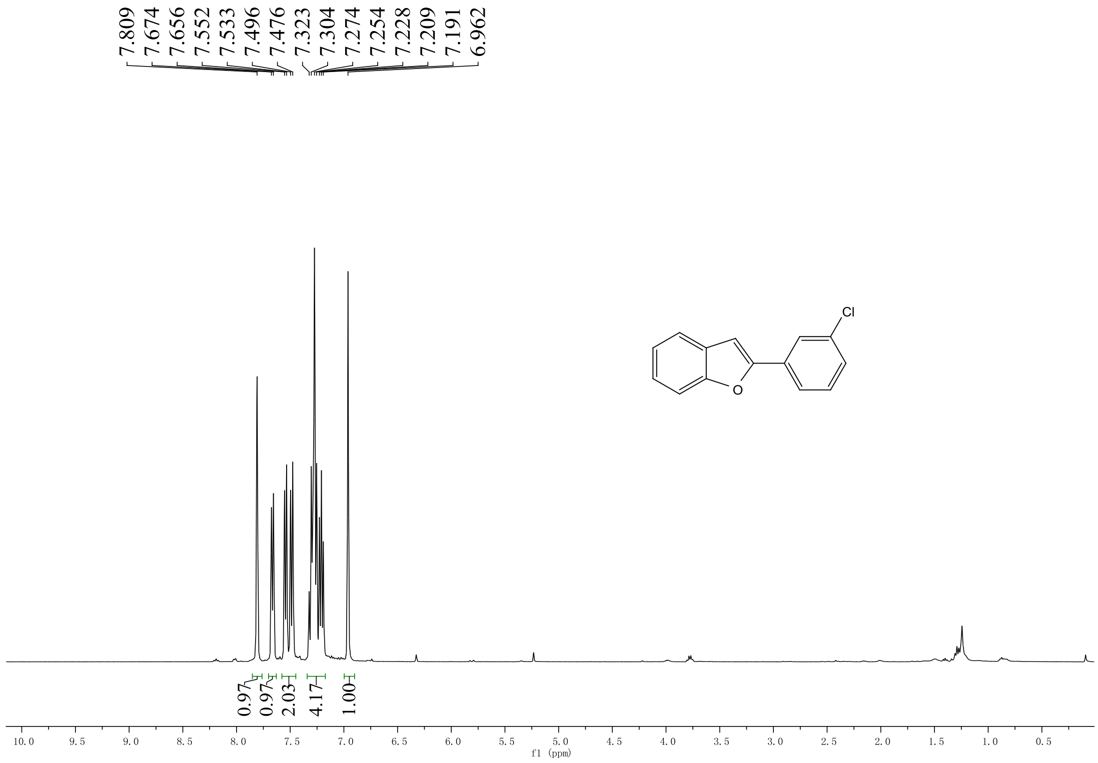
7.809
7.674
7.656
7.552
7.533
7.496
7.476
7.323
7.304
7.274
7.254
7.228
7.209
7.191
6.962
Cl
O
0.97
0.97
2.03
4.17
1.00
f1 (ppm)

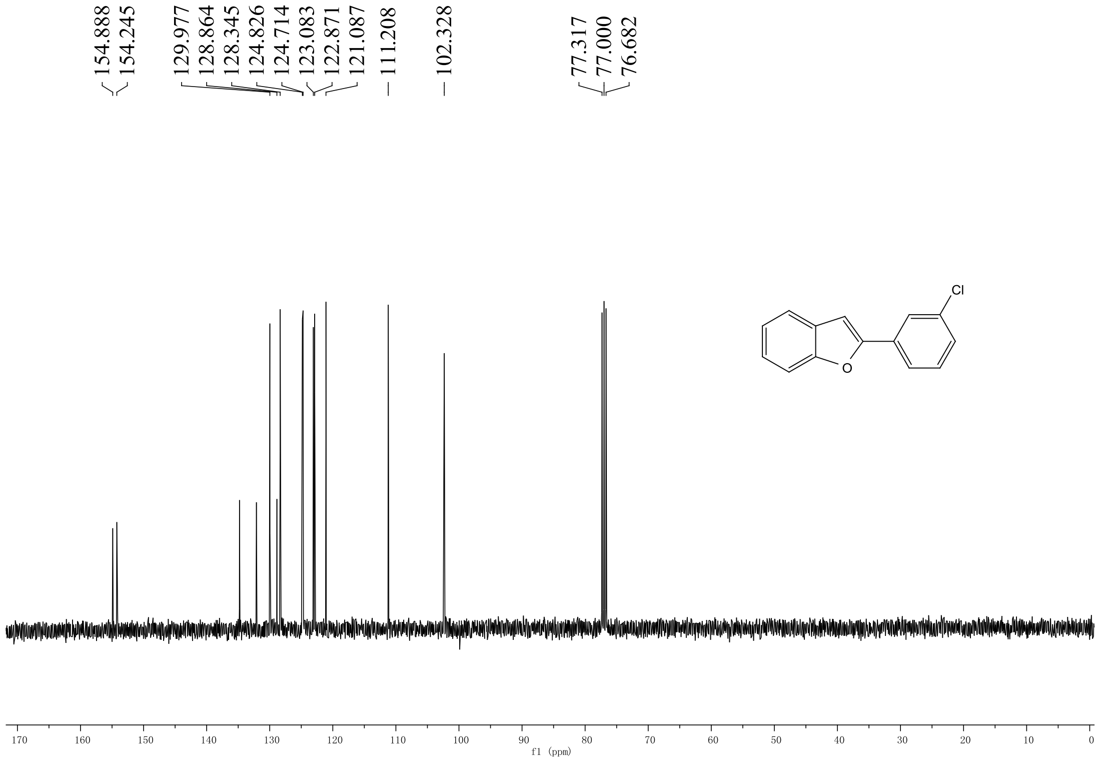
154.888
154.245
129.977
128.864
128.345
124.826
124.714
123.083
122.871
121.087
111.208
102.328
77.317
77.000
76.682
Cl
O
f1 (ppm)

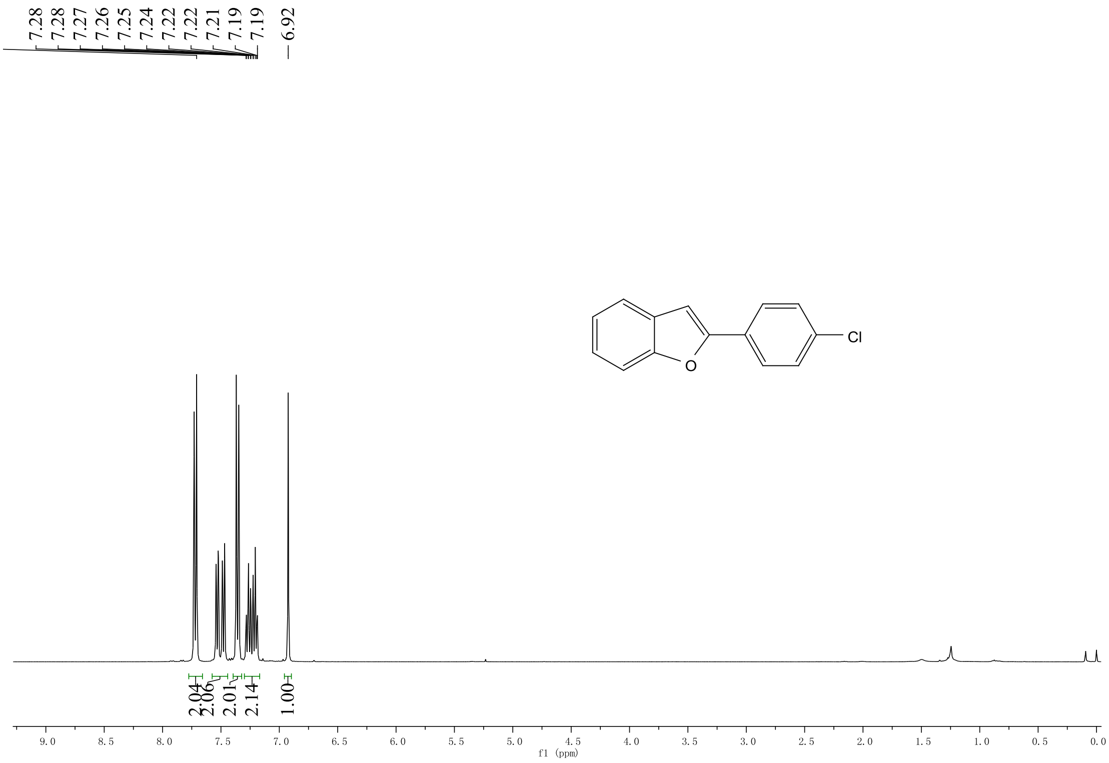
7.28
7.28
7.27
7.26
7.25
7.24
7.22
7.22
7.21
7.19
7.19
6.92
Cl
O
2.04
2.06
2.01
2.14
1.00
f1 (ppm)

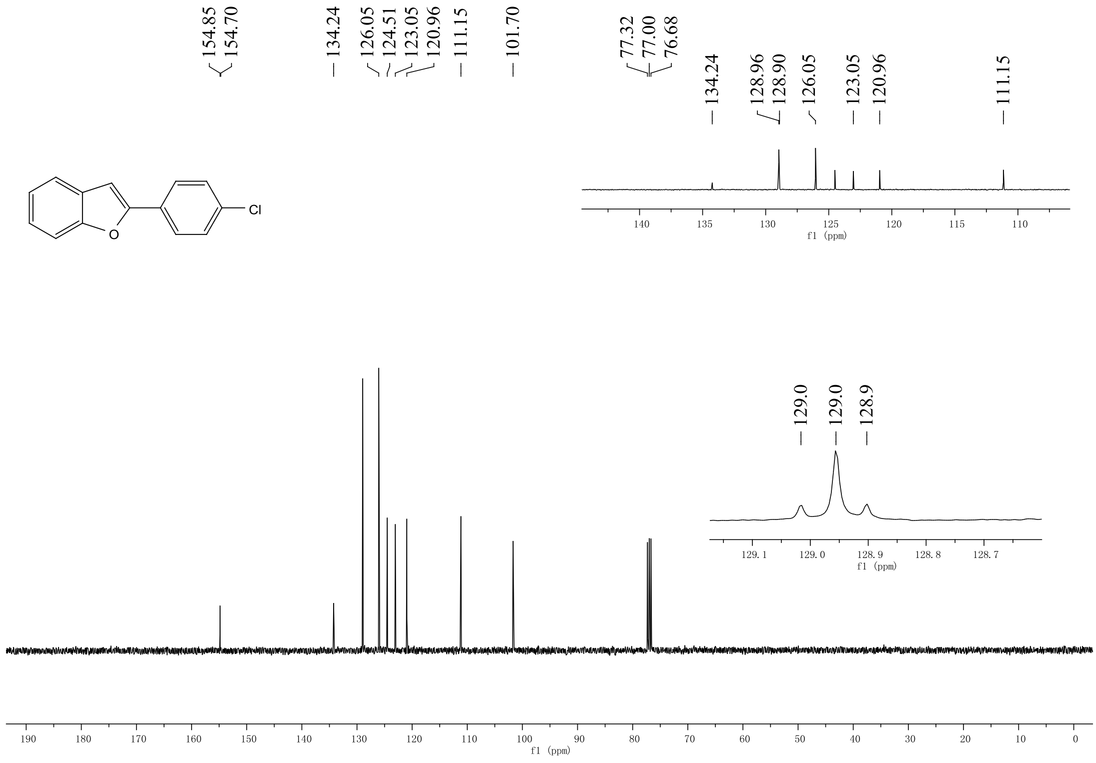
154.85
154.70
134.24
126.05
124.51
123.05
120.96
111.15
101.70
77.32
77.00
76.68
134.24
128.96
128.90
126.05
123.05
120.96
111.15
Cl
O
129.0
129.0
128.9
f1 (ppm)

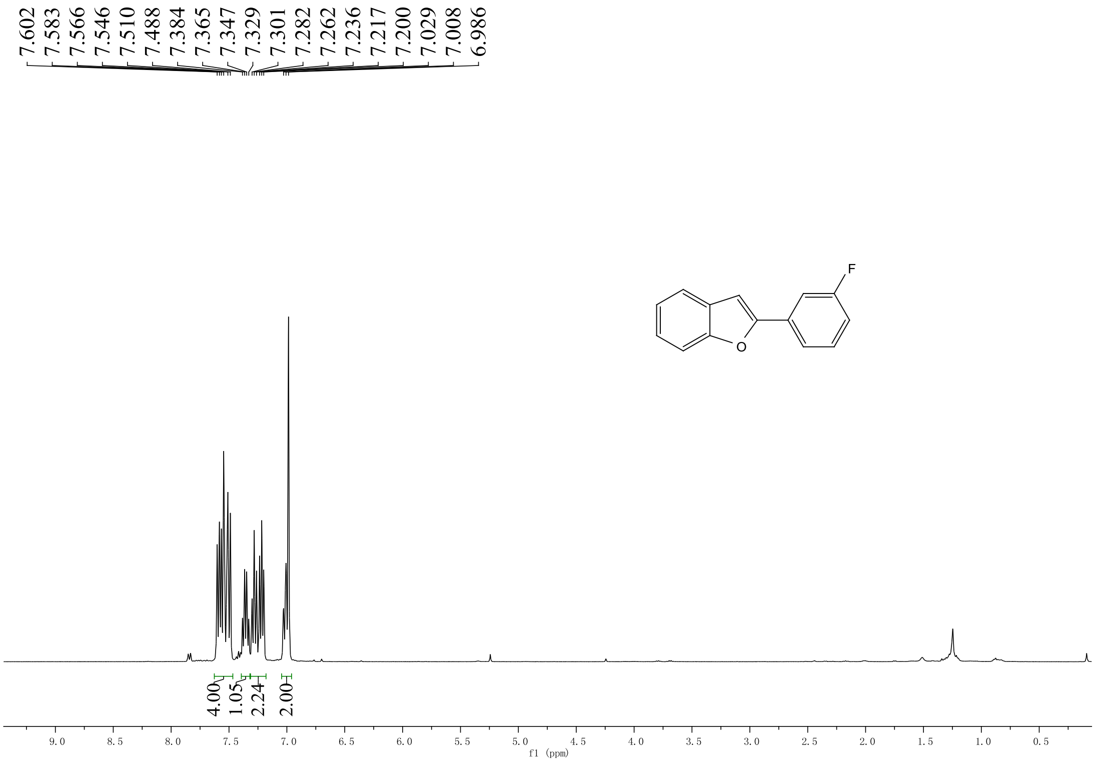
7.602
7.583
7.566
7.546
7.510
7.488
7.384
7.365
7.347
7.329
7.301
7.282
7.262
7.236
7.217
7.200
7.029
7.008
6.986
F
O
4.00
1.05
2.24
2.00
9.0 8.5 8.0 7.5 7.0 6.5 6.0 5.5 5.0 4.5 4.0 3.5 3.0 2.5 2.0 1.5 1.0 0.5
f1 (ppm)

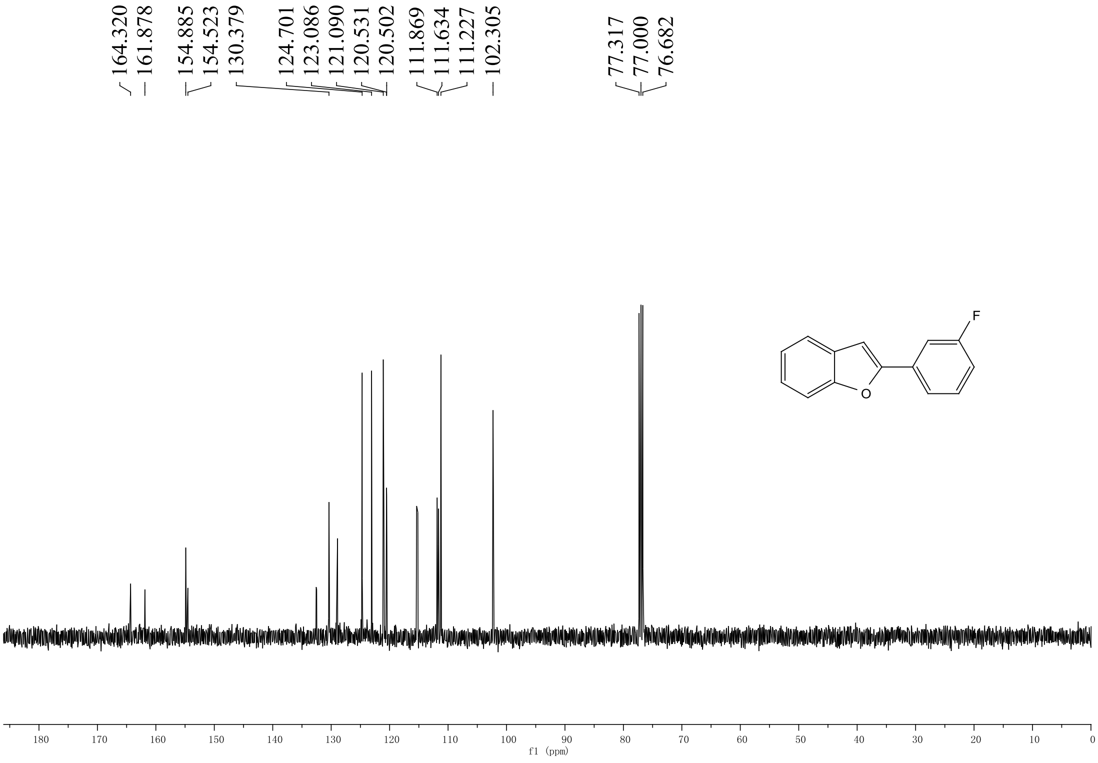
164.320
161.878
154.885
154.523
130.379
124.701
123.086
121.090
120.531
120.502
111.869
111.634
111.227
102.305
77.317
77.000
76.682
F
O
180 170 160 150 140 130 120 110 100 90 80 70 60 50 40 30 20 10 0
f1 (ppm)

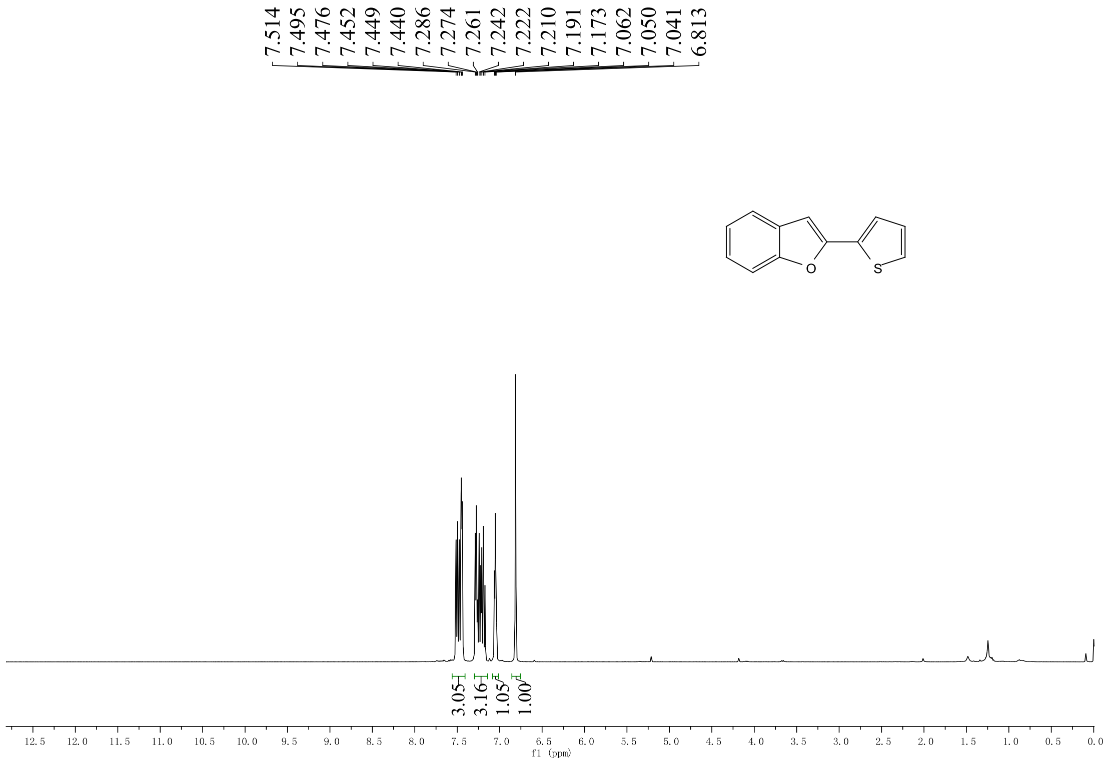
7.514
7.495
7.476
7.452
7.449
7.440
7.286
7.274
7.261
7.242
7.222
7.210
7.191
7.173
7.062
7.050
7.041
6.813
O
S
3.05
3.16
1.05
1.00
f1 (ppm)

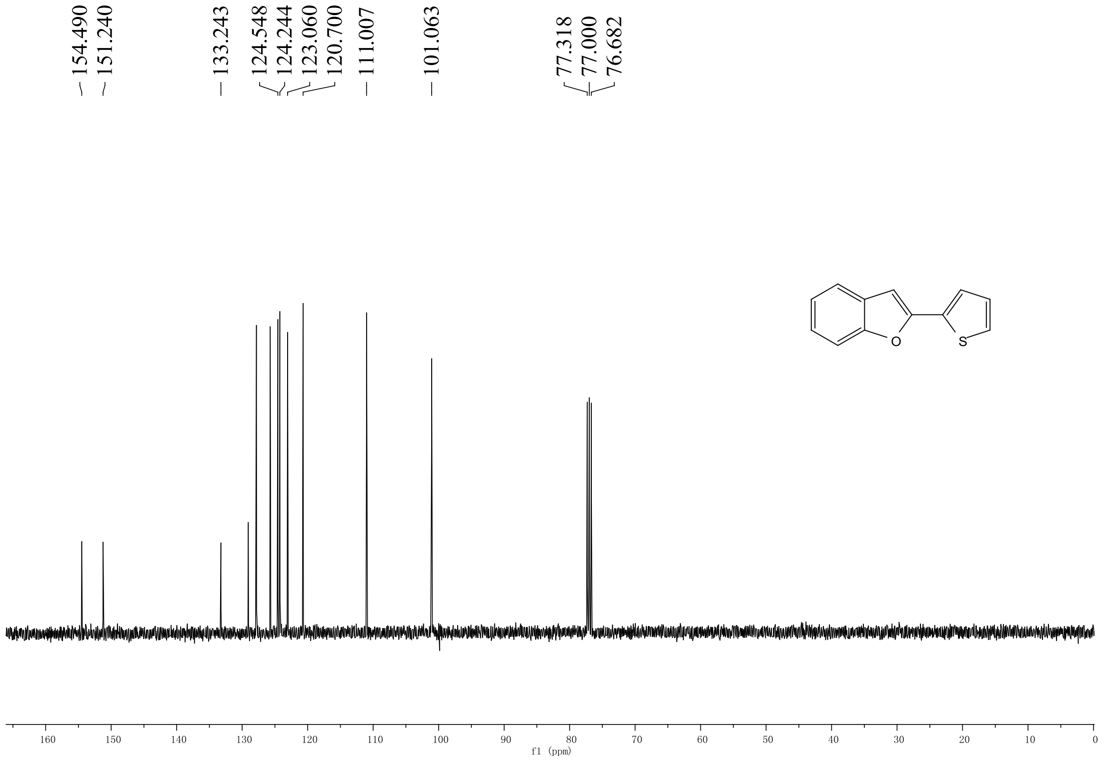
154.490
151.240
133.243
124.548
124.244
123.060
120.700
111.007
101.063
77.318
77.000
76.682
O
S
f1 (ppm)

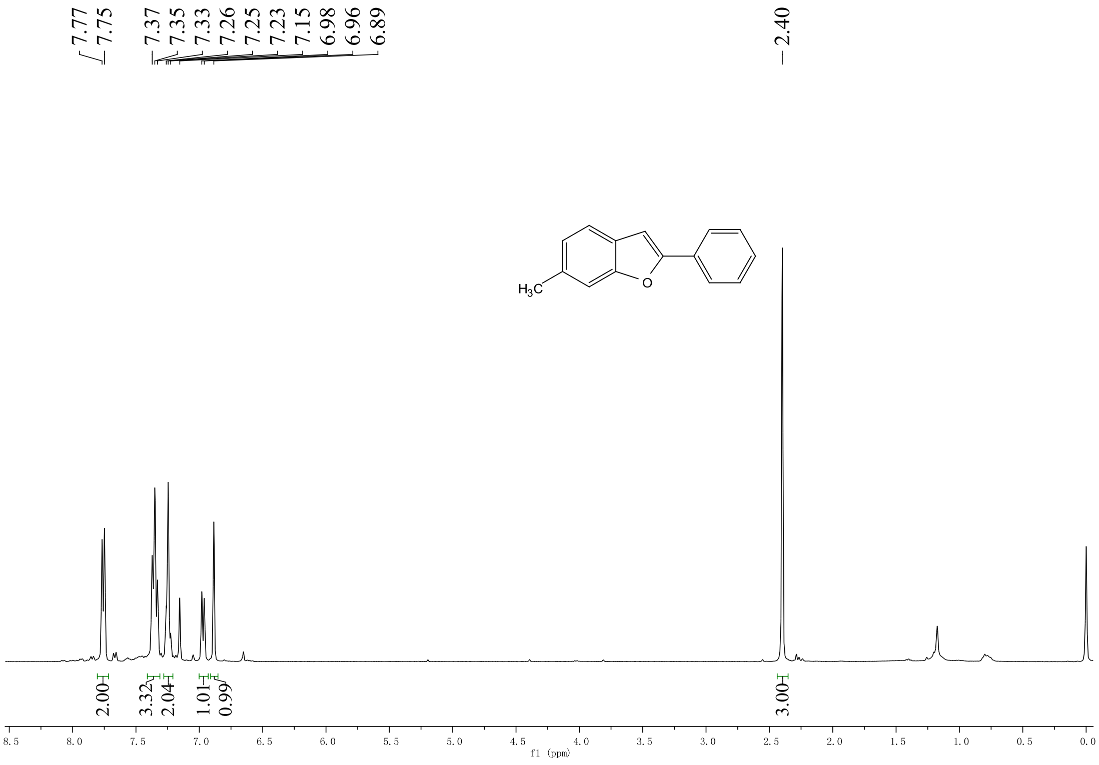
7.77
7.75
7.37
7.35
7.33
7.26
7.25
7.23
7.15
6.98
6.96
6.89
2.40
H_3C
O
2.00
3.32
2.04
1.01
0.99
3.00
8.5 8.0 7.5 7.0 6.5 6.0 5.5 5.0 4.5 4.0 3.5 3.0 2.5 2.0 1.5 1.0 0.5 0.0
f1 (ppm)

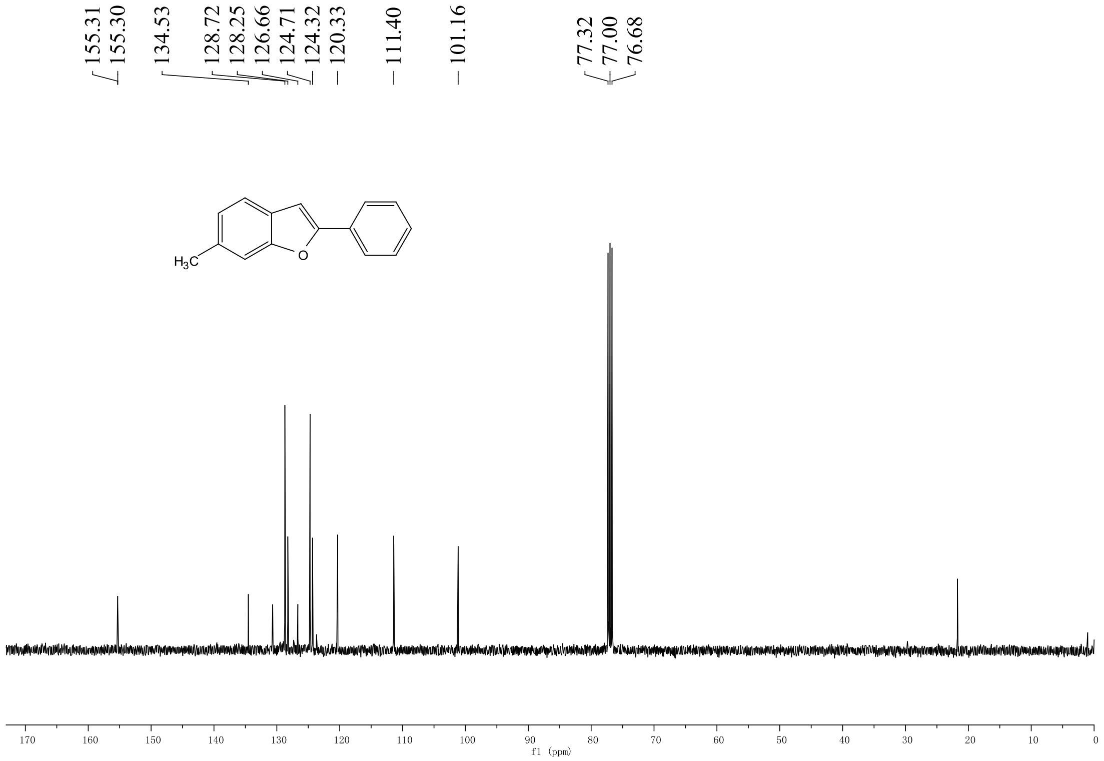
155.31
155.30
134.53
128.72
128.25
126.66
124.71
124.32
120.33
111.40
101.16
77.32
77.00
76.68
H_3C
O
170 160 150 140 130 120 110 100 90 80 70 60 50 40 30 20 10 0
f1 (ppm)

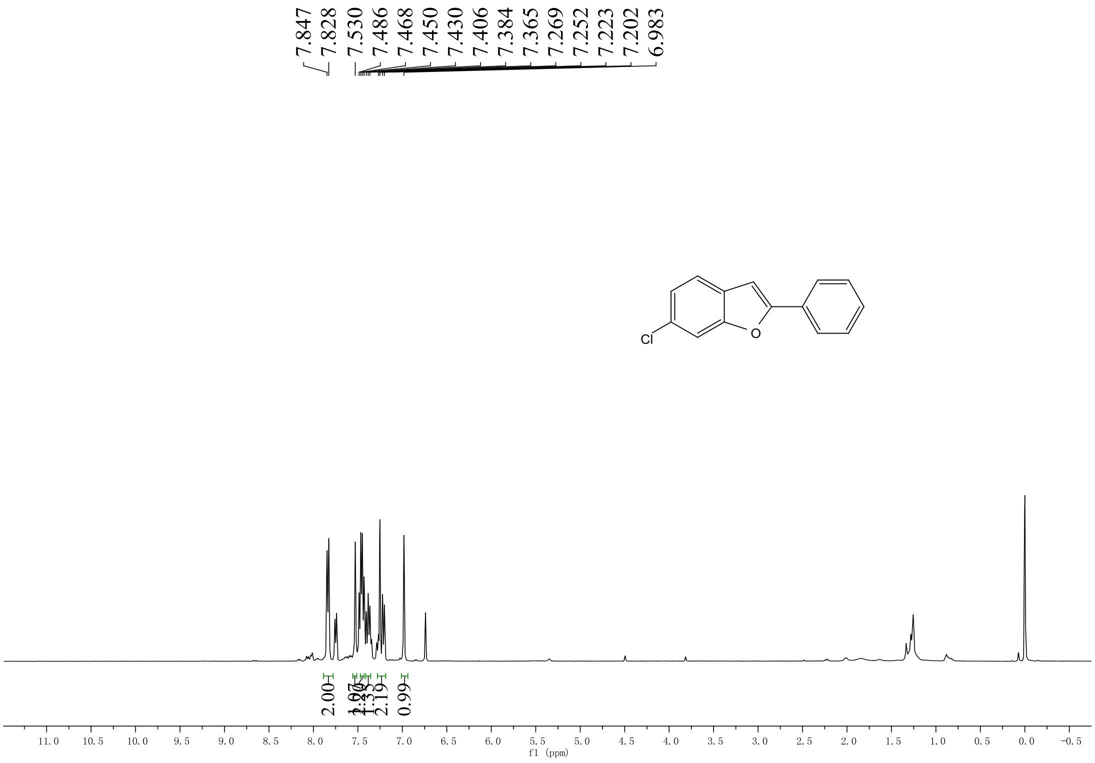
7.847
7.828
7.530
7.486
7.468
7.450
7.430
7.406
7.384
7.365
7.269
7.252
7.223
7.202
6.983
Cl
O
2.00
1.07
2.19
0.99
f1 (ppm)

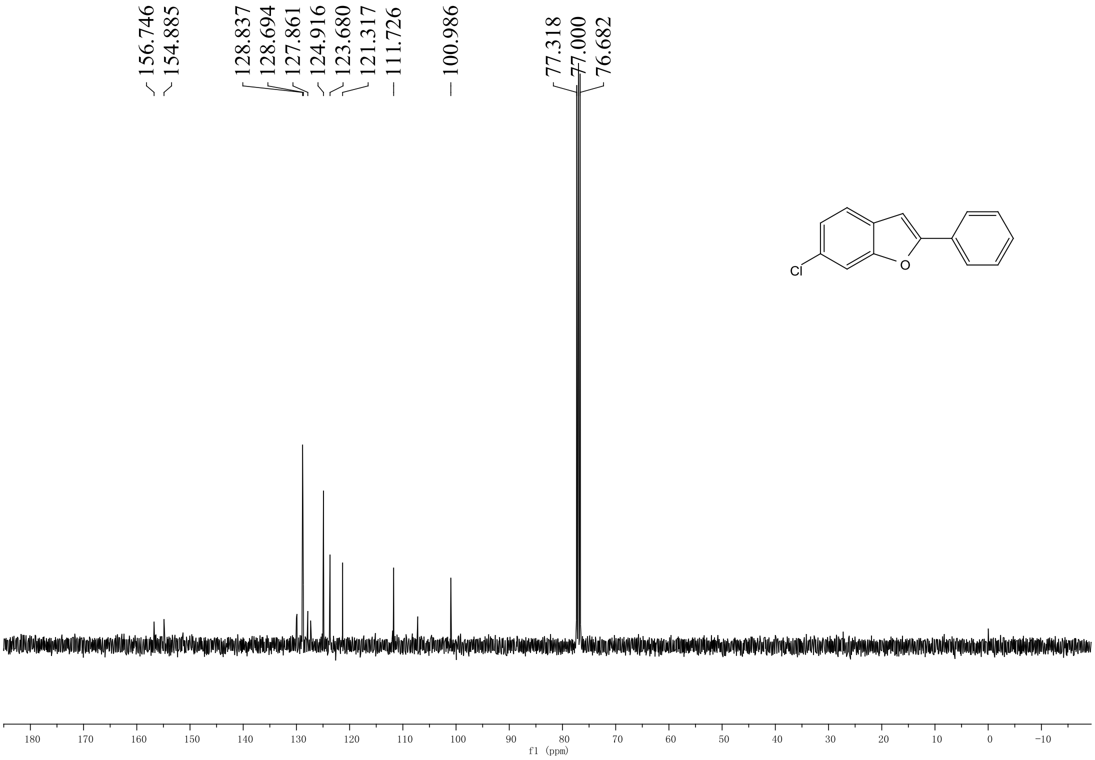
156.746
154.885
128.837
128.694
127.861
124.916
123.680
121.317
111.726
100.986
77.318
77.000
76.682
Cl
O
f1 (ppm)

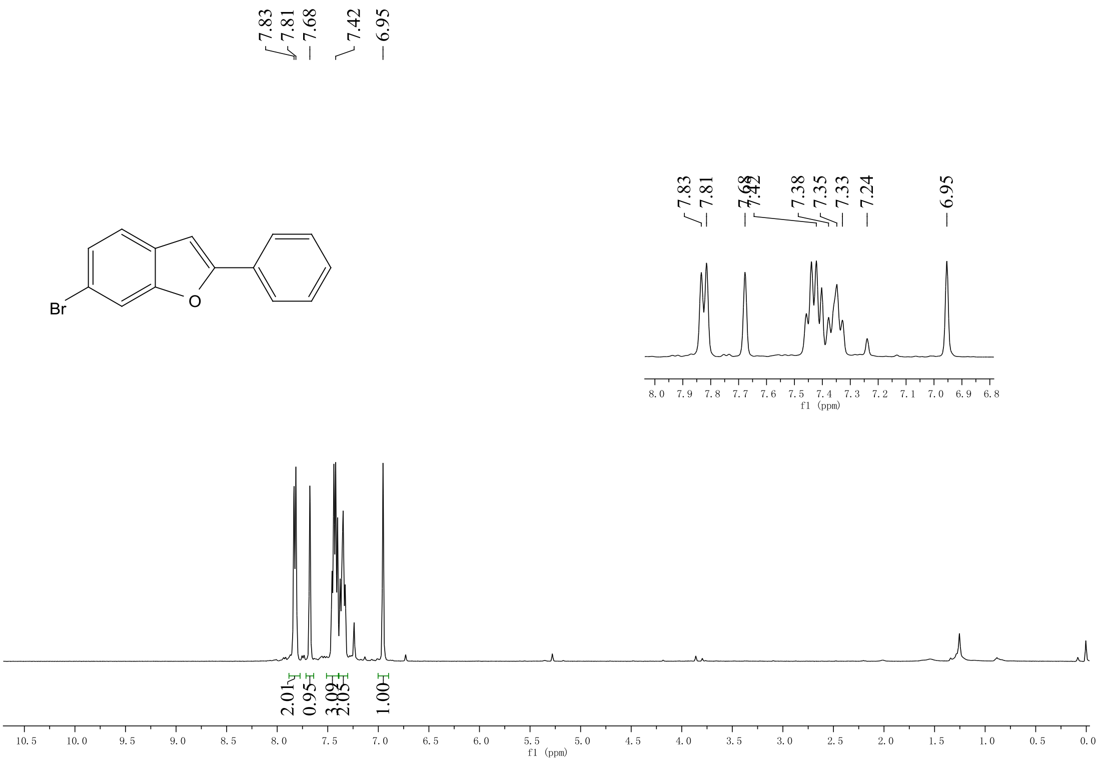
7.83
7.81
7.68
7.42
6.95
7.38
7.35
7.33
7.24
Br
O
2.01
0.95
3.02
2.05
1.00
f1 (ppm)

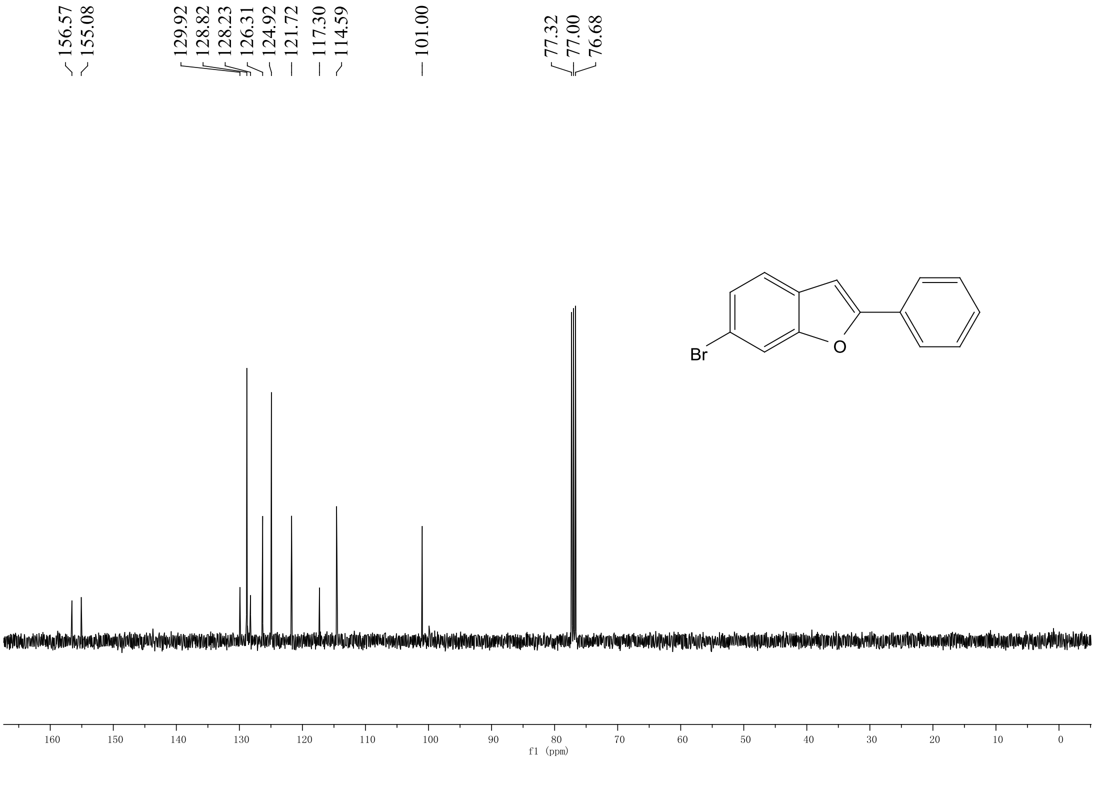
156.57
155.08
129.92
128.82
128.23
126.31
124.92
121.72
117.30
114.59
101.00
77.32
77.00
76.68
Br
O
f1 (ppm)

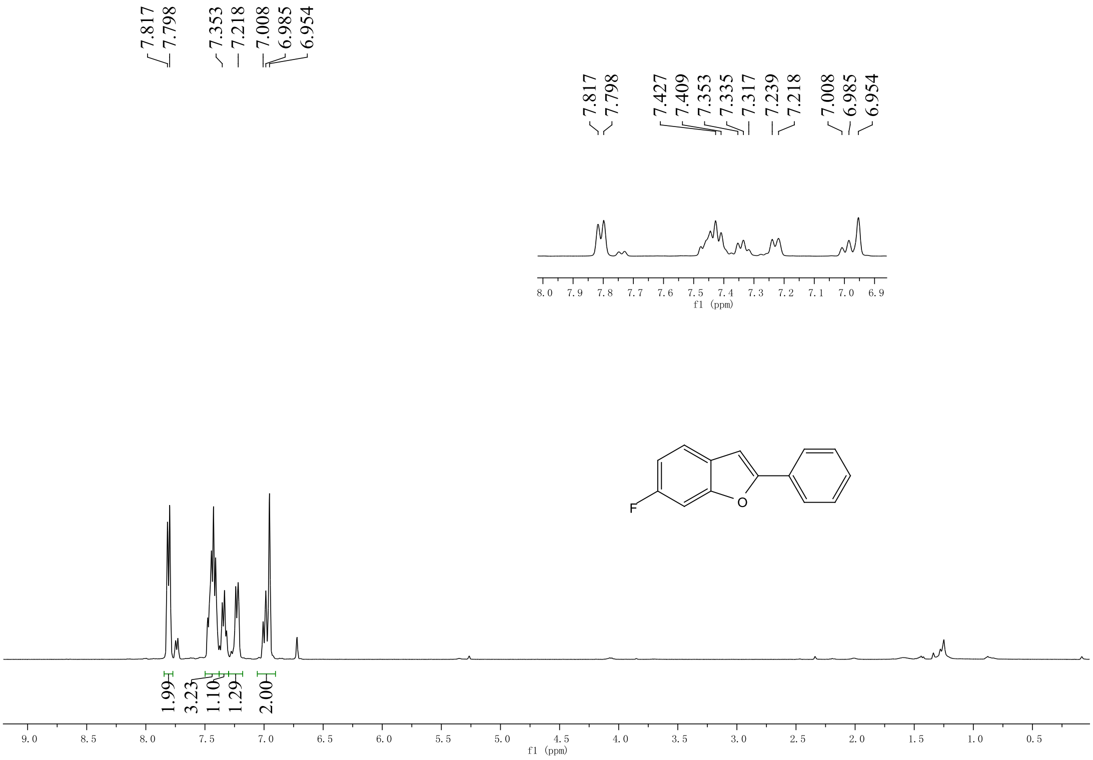
7.817
7.798
7.353
7.218
7.008
6.985
6.954
7.817
7.798
7.427
7.409
7.353
7.335
7.317
7.239
7.218
7.008
6.985
6.954
f1 (ppm)
F
O
1.99
3.23
1.10
1.29
2.00
f1 (ppm)

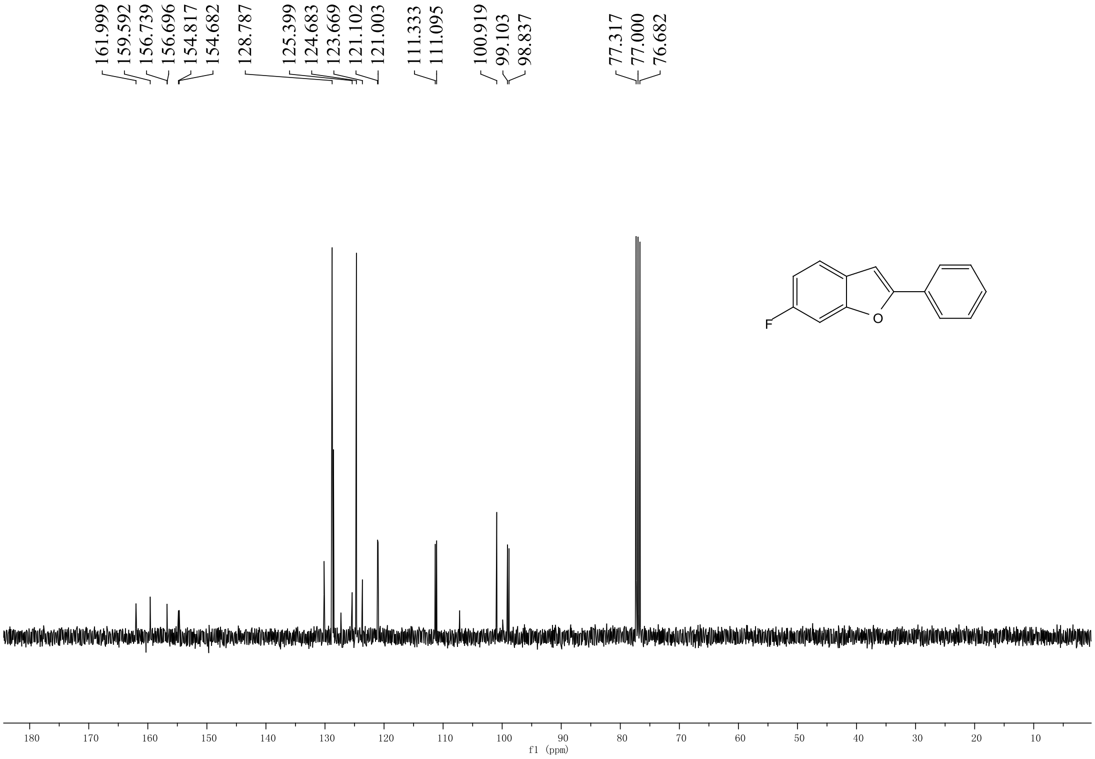
161.999
159.592
156.739
156.696
154.817
154.682
128.787
125.399
124.683
123.669
121.102
121.003
111.333
111.095
100.919
99.103
98.837
77.317
77.000
76.682
F
O
f1 (ppm)

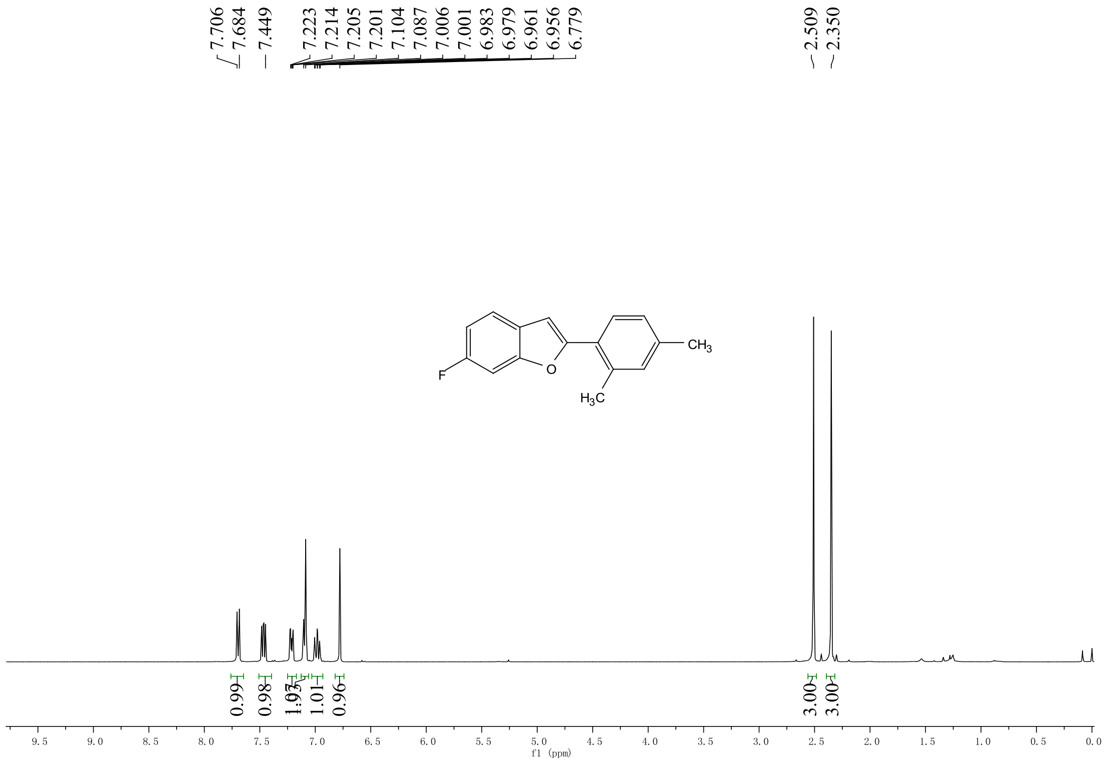
7.706
7.684
7.449
7.223
7.214
7.205
7.201
7.104
7.087
7.006
7.001
6.983
6.979
6.961
6.956
6.779
2.509
2.350
CH3
F
O
H3C
0.99
0.98
1.07
1.95
1.01
0.96
3.00
3.00
f1 (ppm)

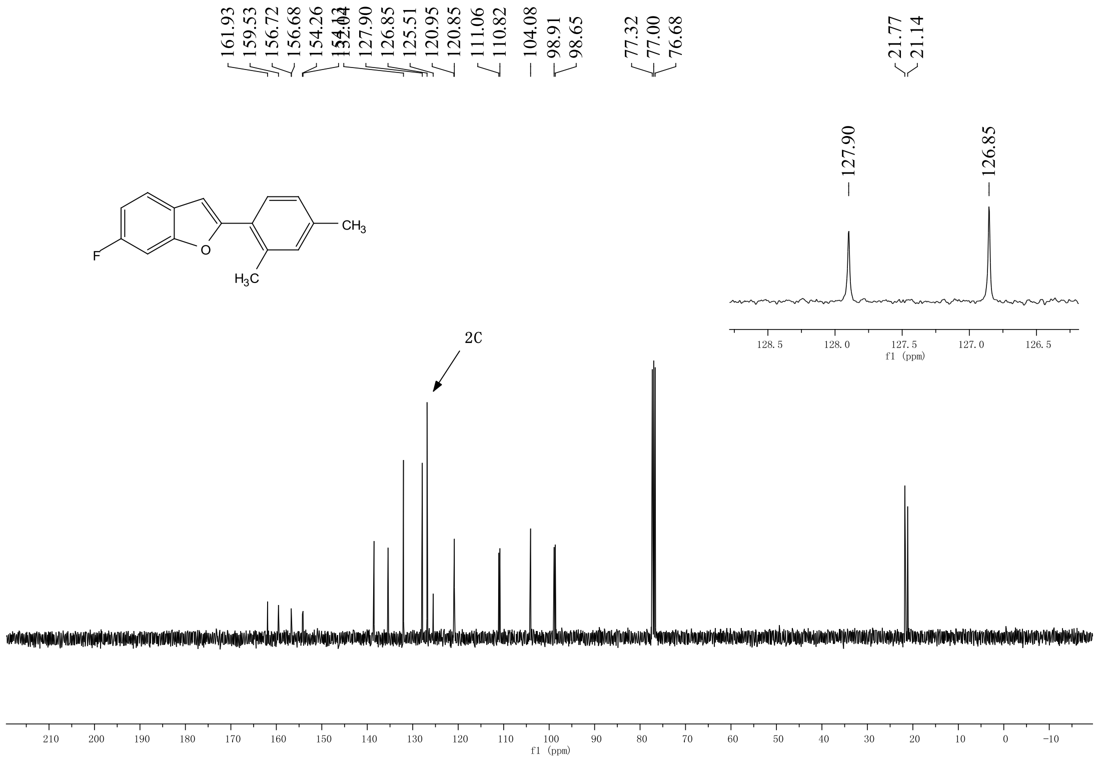
161.93
159.53
156.72
156.68
154.26
154.12
152.04
127.90
126.85
125.51
120.95
120.85
111.06
110.82
104.08
98.91
98.65
77.32
77.00
76.68
21.77
21.14
127.90
126.85
CH3
F
O
H3C
2C
f1 (ppm)

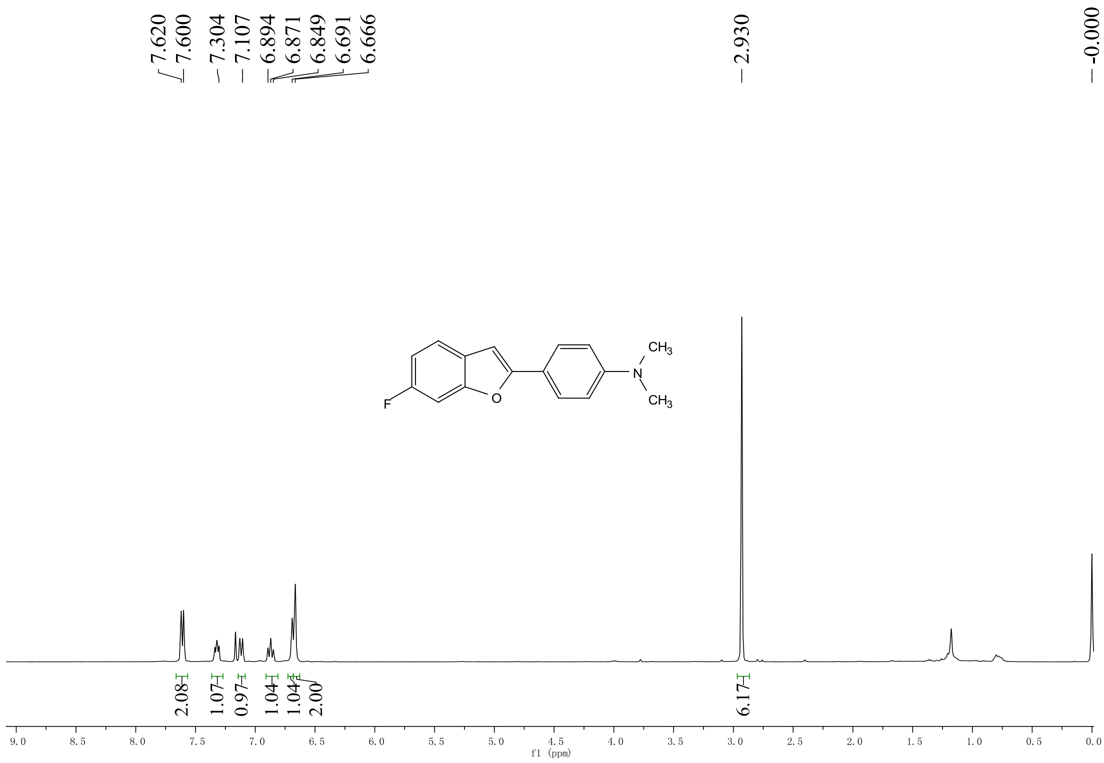
7.620
7.600
7.304
7.107
6.894
6.871
6.849
6.691
6.666
2.930
-0.000
CH3
N
CH3
F
O
2.08
1.07
0.97
1.04
1.04
2.00
6.17
9.0 8.5 8.0 7.5 7.0 6.5 6.0 5.5 5.0 4.5 4.0 3.5 3.0 2.5 2.0 1.5 1.0 0.5 0.0
f1 (ppm)

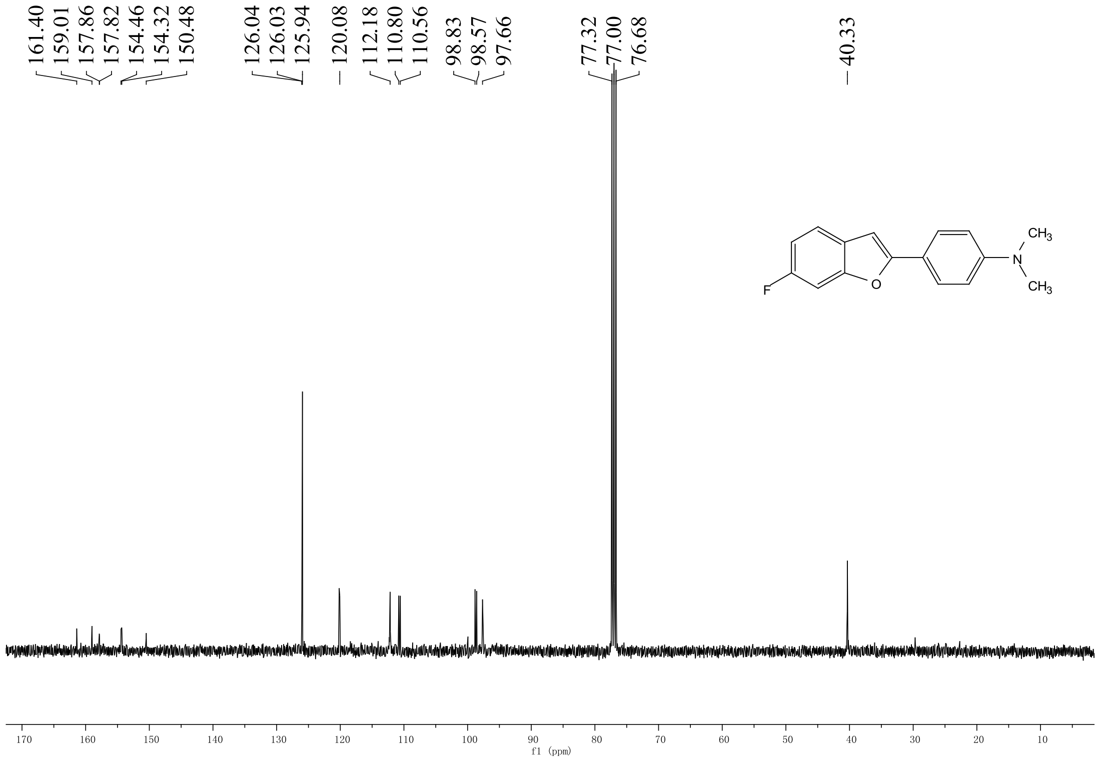
161.40
159.01
157.86
157.82
154.46
154.32
150.48
126.04
126.03
125.94
120.08
112.18
110.80
110.56
98.83
98.57
97.66
77.32
77.00
76.68
40.33
CH3
N
CH3
F
O
170 160 150 140 130 120 110 100 90 80 70 60 50 40 30 20 10
f1 (ppm)

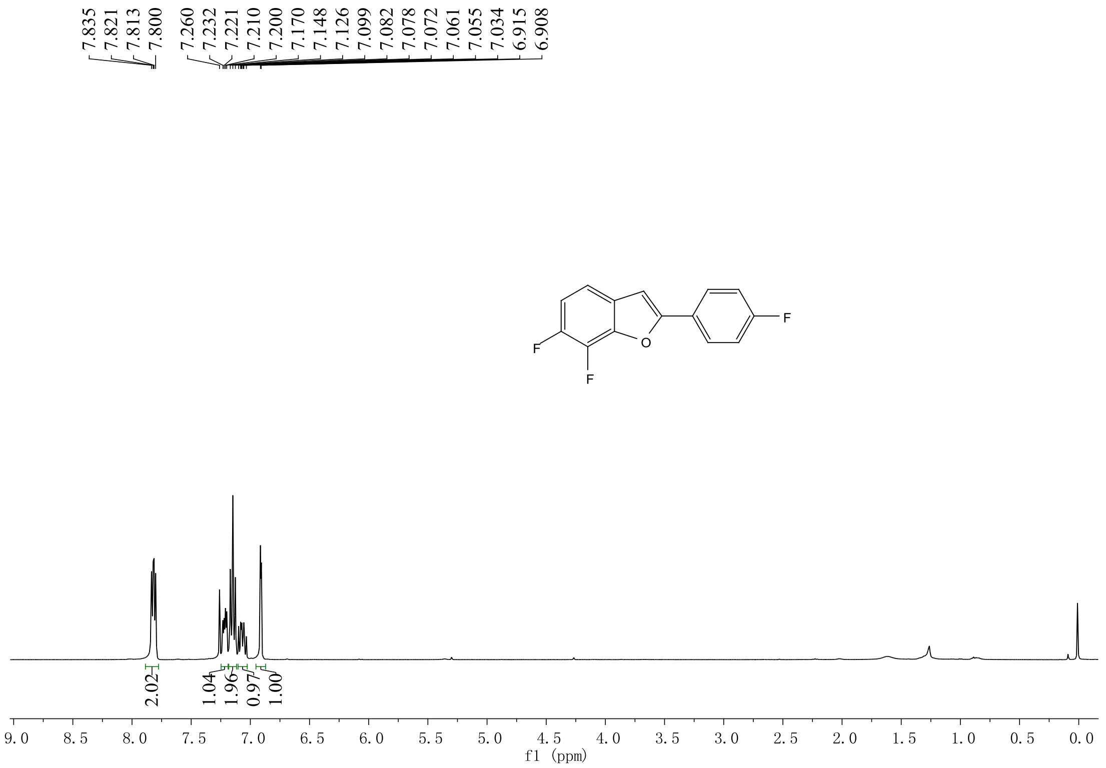
7.835
7.821
7.813
7.800
7.260
7.232
7.221
7.210
7.200
7.170
7.148
7.126
7.099
7.082
7.078
7.072
7.061
7.055
7.034
6.915
6.908
F
O
F
F
2.02
1.04
1.96
0.97
1.00
f1 (ppm)

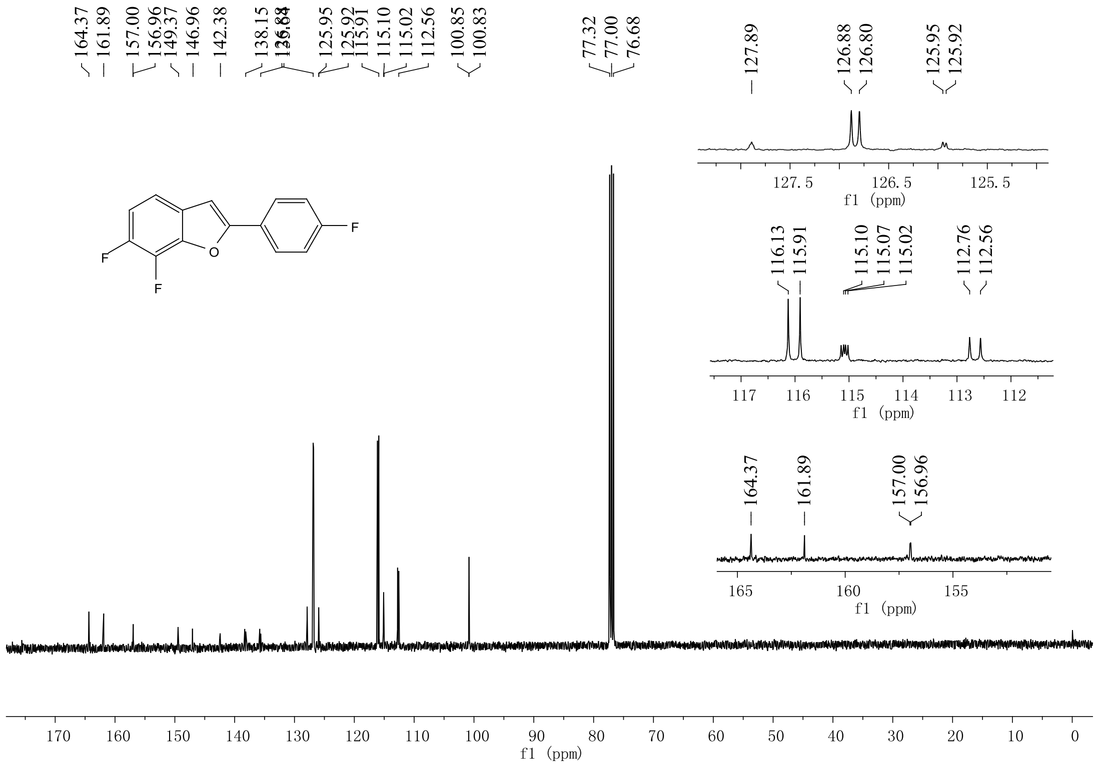
164.37
161.89
157.00
156.96
149.37
146.96
142.38
138.15
125.95
125.92
115.91
115.10
115.02
112.56
100.85
100.83
77.32
77.00
76.68
127.89
126.88
126.80
125.95
125.92
116.13
115.91
115.10
115.07
115.02
112.76
112.56
164.37
161.89
157.00
156.96
F
O
F
F
f1 (ppm)

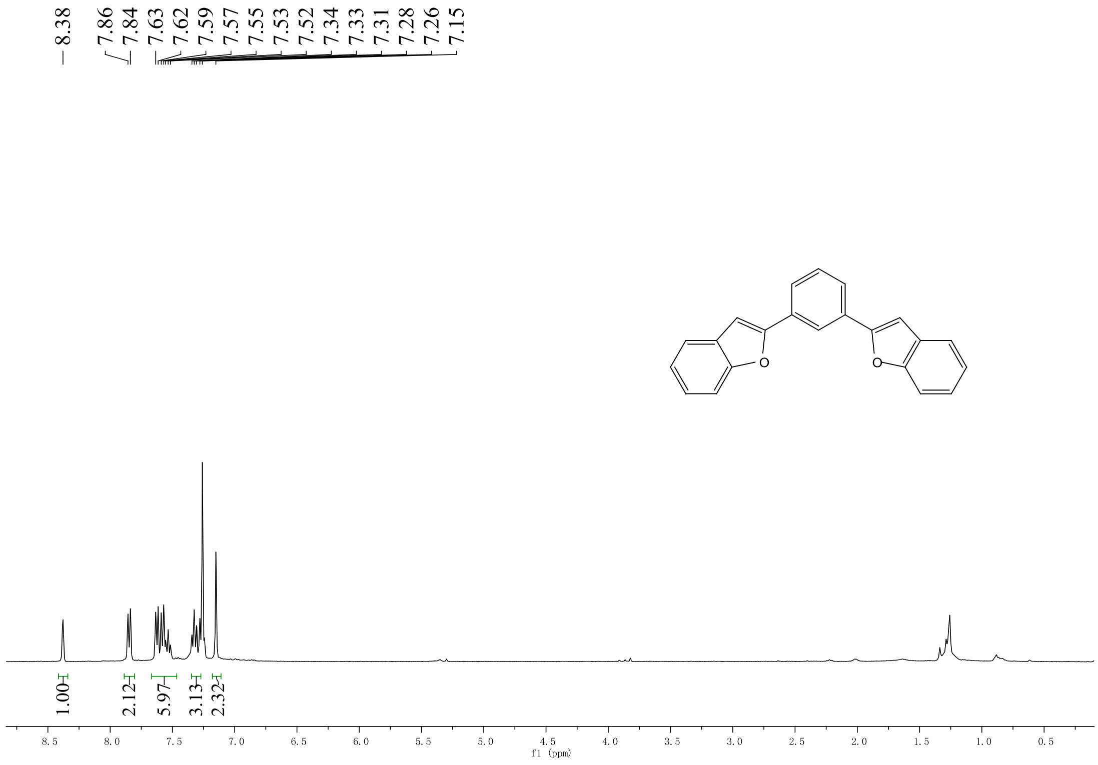
8.38
7.86
7.84
7.63
7.62
7.59
7.57
7.55
7.53
7.52
7.34
7.33
7.31
7.28
7.26
7.15
O
O
1.00
2.12
5.97
3.13
2.32
8.5 8.0 7.5 7.0 6.5 6.0 5.5 5.0 4.5 4.0 3.5 3.0 2.5 2.0 1.5 1.0 0.5
f1 (ppm)

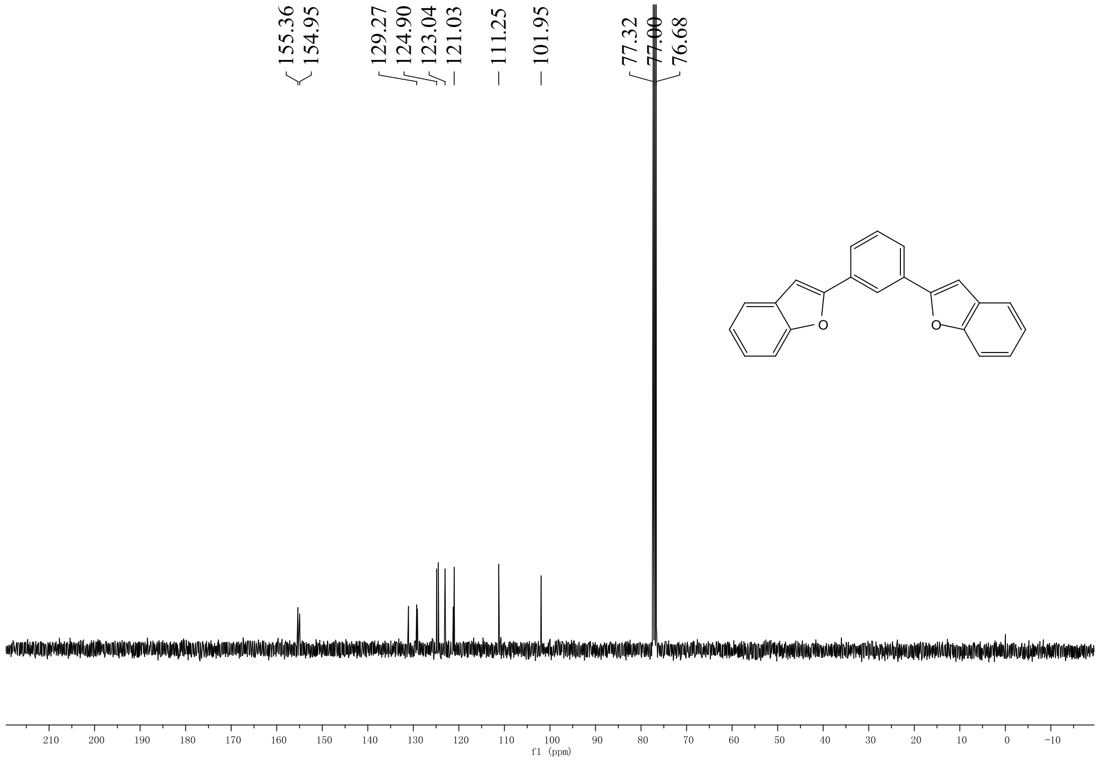
155.36
154.95
129.27
124.90
123.04
121.03
111.25
101.95
77.32
77.00
76.68
O
O
210 200 190 180 170 160 150 140 130 120 110 100 90 80 70 60 50 40 30 20 10 0 -10
f1 (ppm)

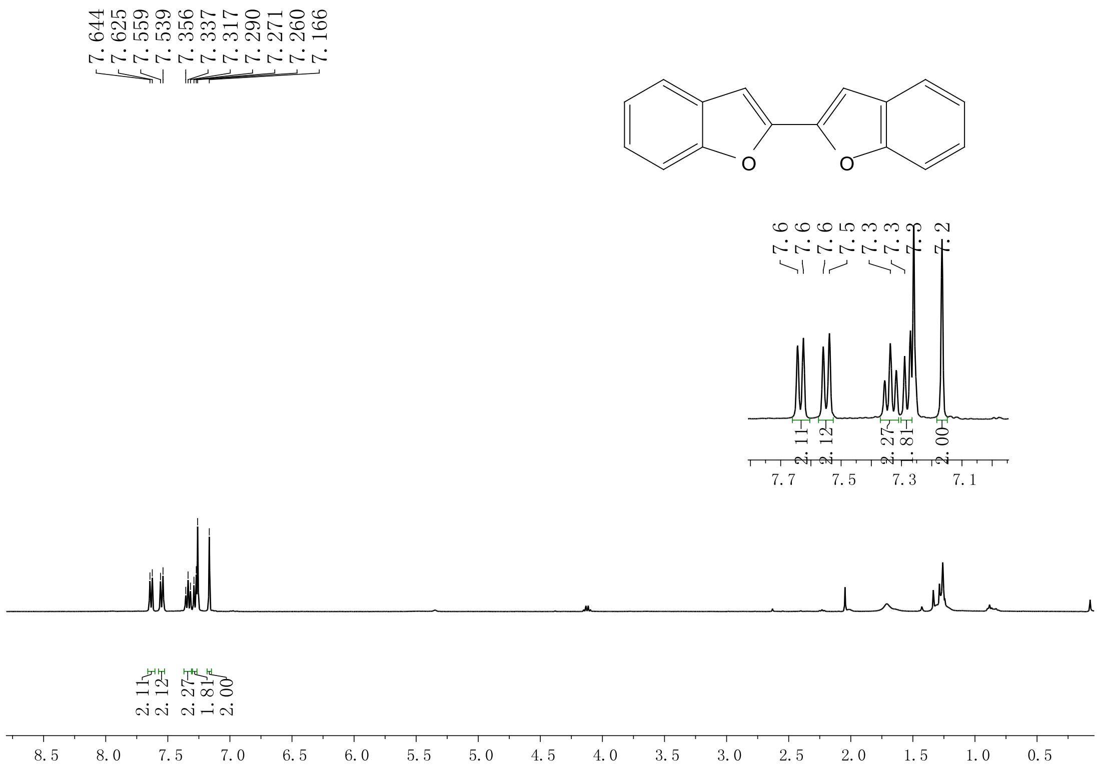
7.644
7.625
7.559
7.539
7.356
7.337
7.317
7.290
7.271
7.260
7.166
O
O
2.11
2.12
2.27
1.81
2.00
8.5 8.0 7.5 7.0 6.5 6.0 5.5 5.0 4.5 4.0 3.5 3.0 2.5 2.0 1.5 1.0 0.5

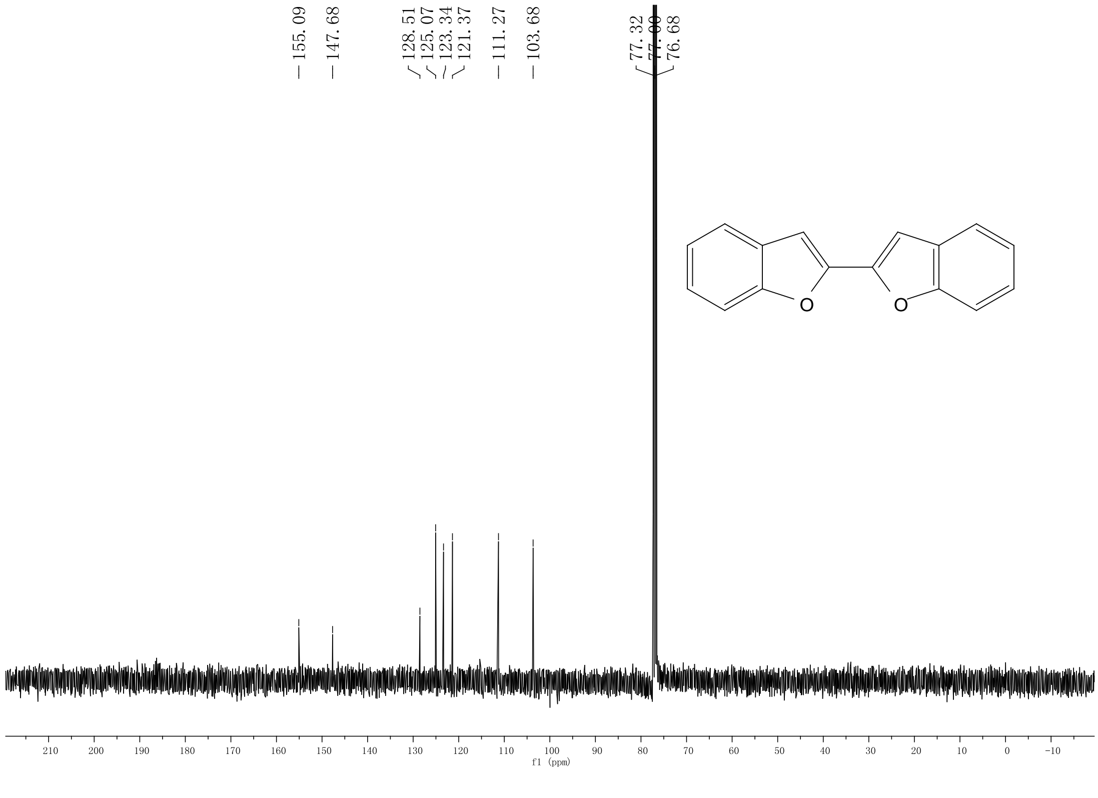
155.09
147.68
128.51
125.07
123.34
121.37
111.27
103.68
77.32
77.00
76.68
O
O
210 200 190 180 170 160 150 140 130 120 110 100 90 80 70 60 50 40 30 20 10 0 -10
f1 (ppm)

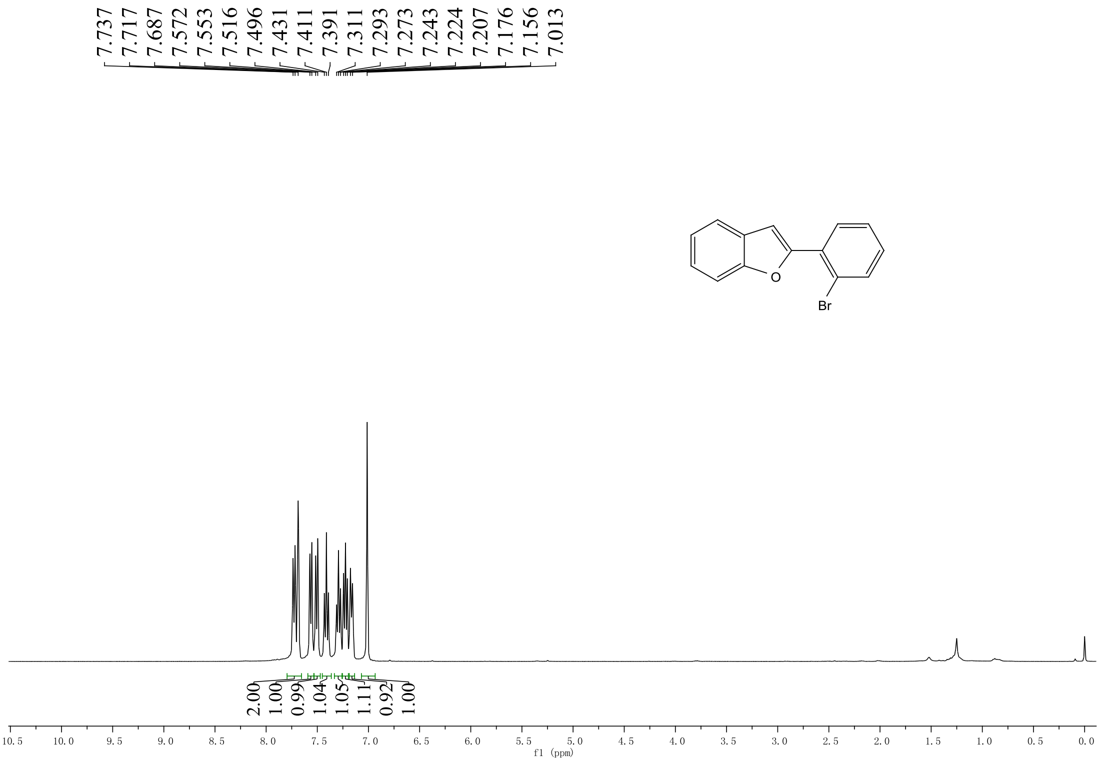
7.737
7.717
7.687
7.572
7.553
7.516
7.496
7.431
7.411
7.391
7.311
7.293
7.273
7.243
7.224
7.207
7.176
7.156
7.013
O
Br
2.00
1.00
0.99
1.04
1.05
1.11
0.92
1.00
f1 (ppm)

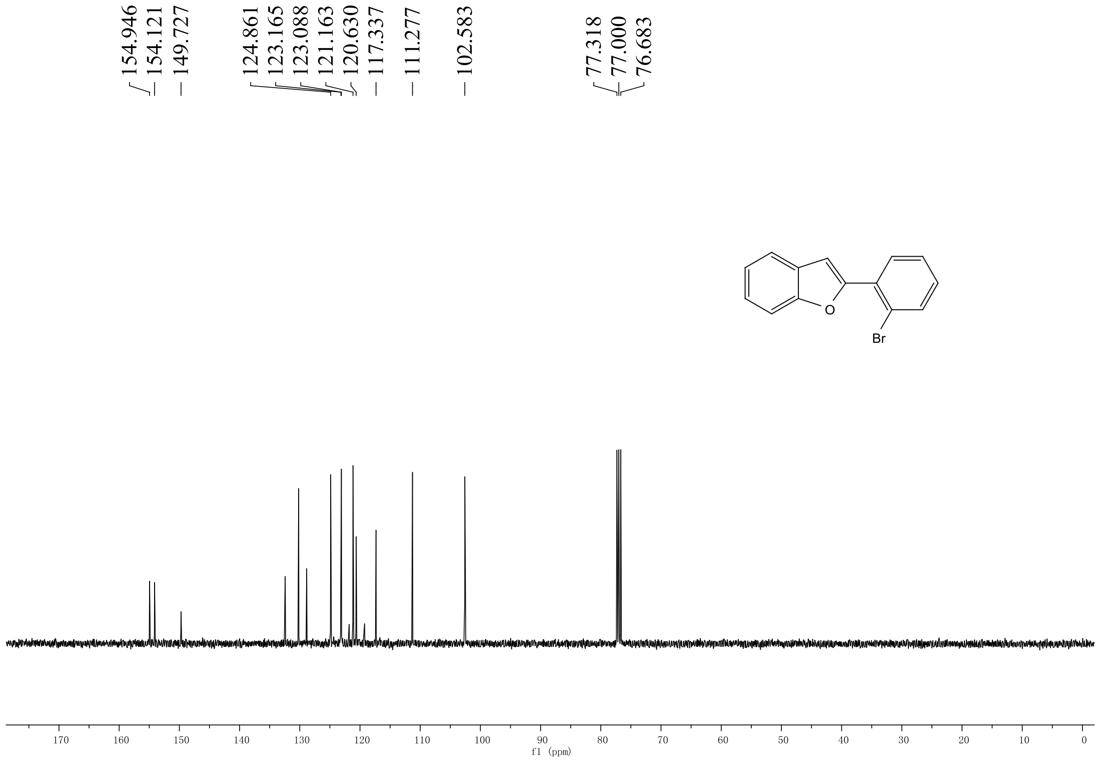
154.946
154.121
149.727
124.861
123.165
123.088
121.163
120.630
117.337
111.277
102.583
77.318
77.000
76.683
O
Br
f1 (ppm)

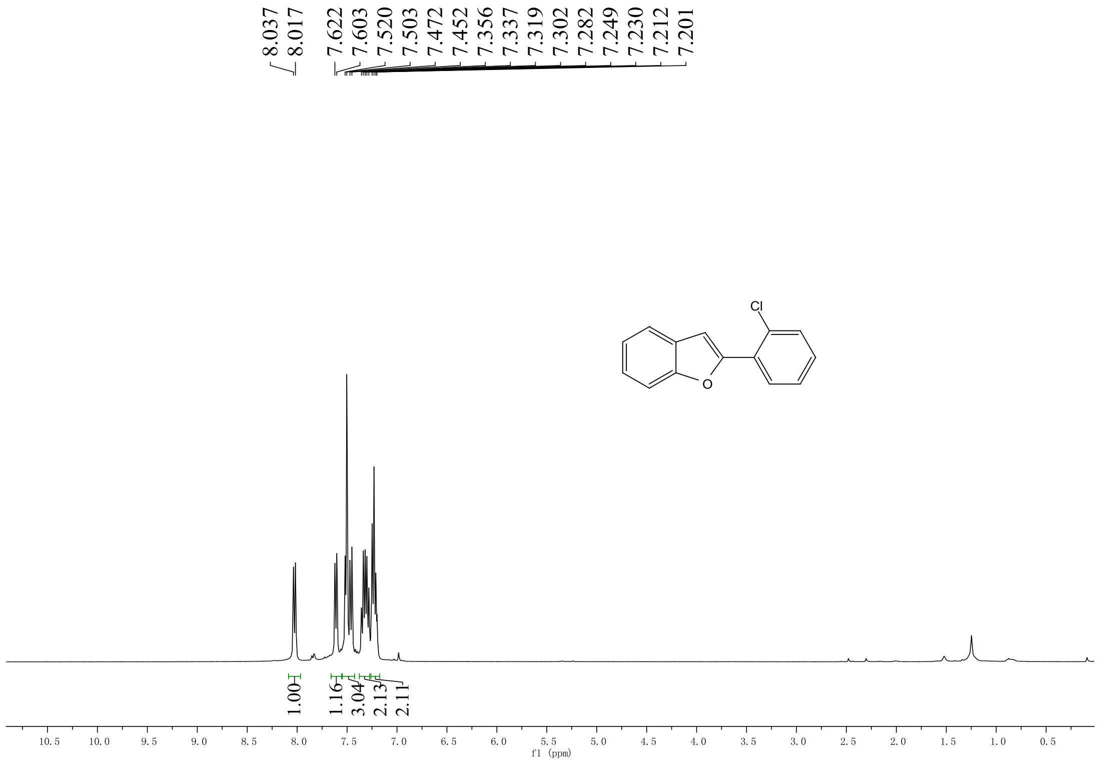
8.037
8.017
7.622
7.603
7.520
7.503
7.472
7.452
7.356
7.337
7.319
7.302
7.282
7.249
7.230
7.212
7.201
Cl
O
1.00
1.16
3.04
2.13
2.11
f1 (ppm)

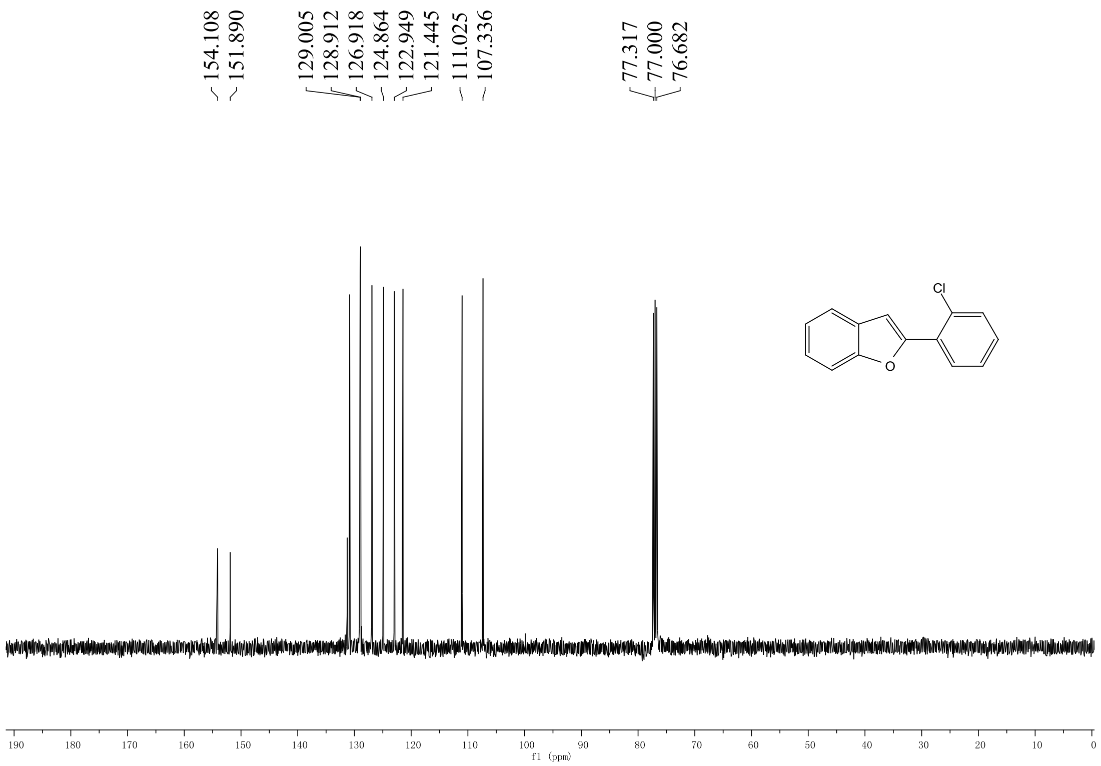
154.108
151.890
129.005
128.912
126.918
124.864
122.949
121.445
111.025
107.336
77.317
77.000
76.682
Cl
O
f1 (ppm)

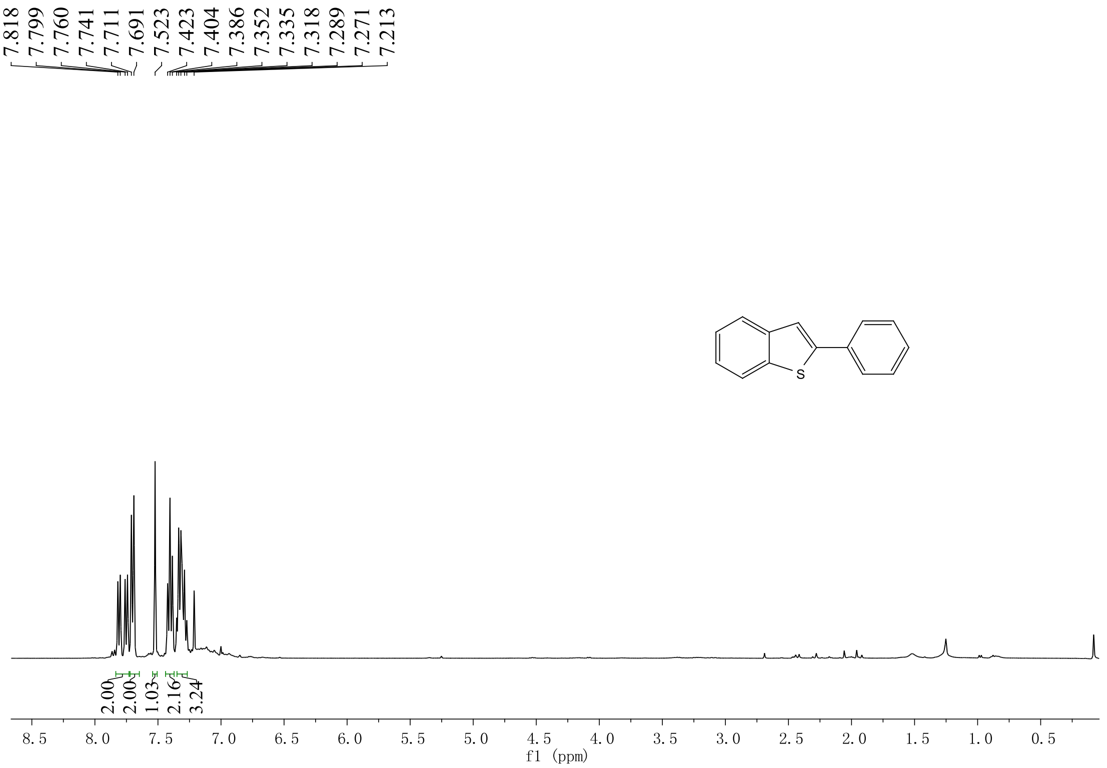
7.818
7.799
7.760
7.741
7.711
7.691
7.523
7.423
7.404
7.386
7.352
7.335
7.318
7.289
7.271
7.213
S
2.00
2.00
1.03
2.16
3.24
8.5 8.0 7.5 7.0 6.5 6.0 5.5 5.0 4.5 4.0 3.5 3.0 2.5 2.0 1.5 1.0 0.5
f1 (ppm)

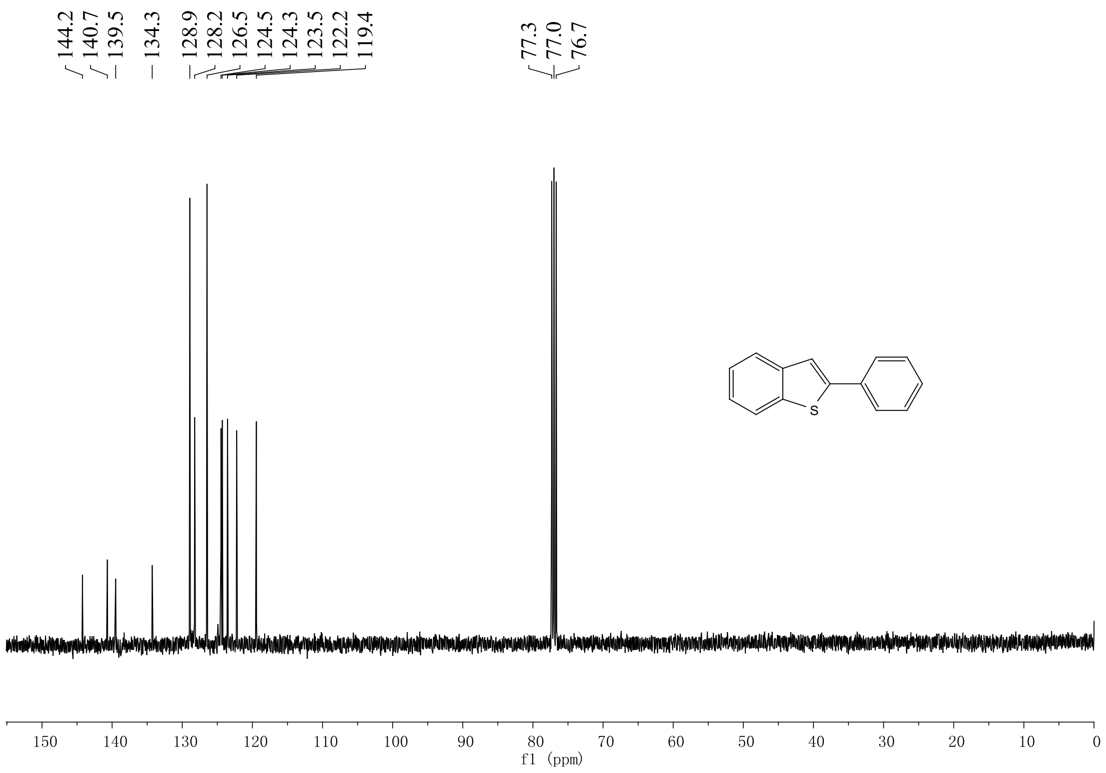
144.2
140.7
139.5
134.3
128.9
128.2
126.5
124.5
124.3
123.5
122.2
119.4
77.3
77.0
76.7
S
150 140 130 120 110 100 90 80 70 60 50 40 30 20 10 0
f1 (ppm)

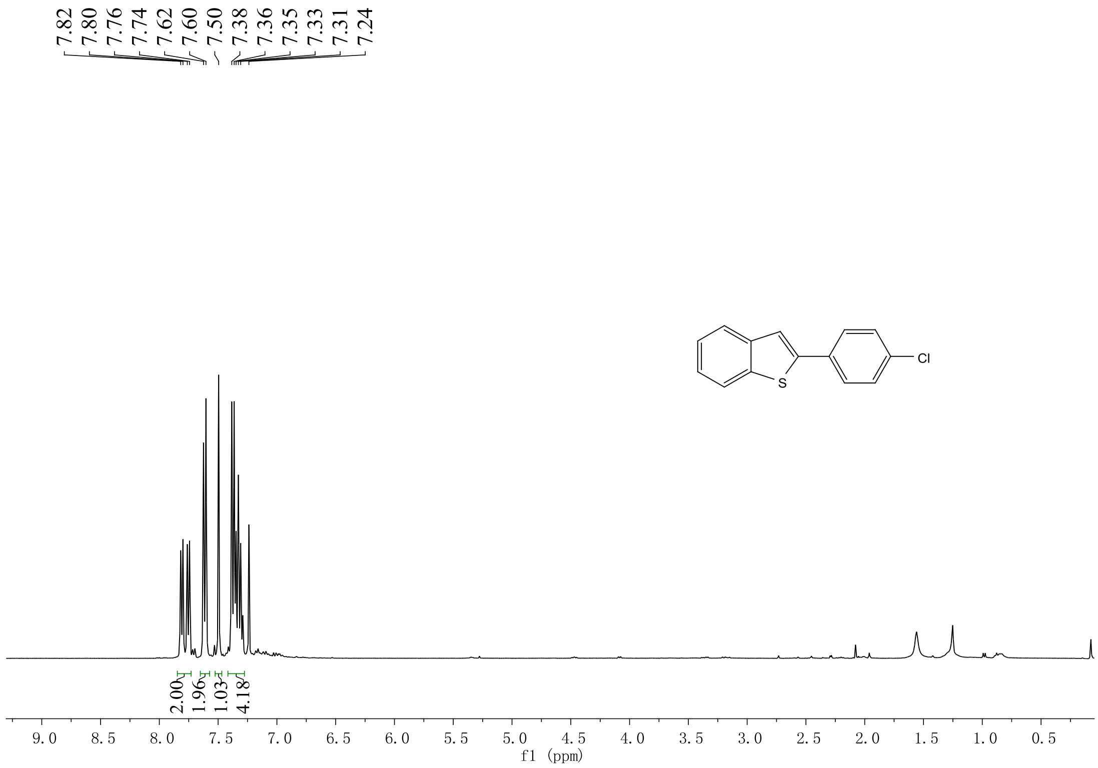
7.82
7.80
7.76
7.74
7.62
7.60
7.50
7.38
7.36
7.35
7.33
7.31
7.24
S
Cl
2.00
1.96
1.03
4.18
9.0 8.5 8.0 7.5 7.0 6.5 6.0 5.5 5.0 4.5 4.0 3.5 3.0 2.5 2.0 1.5 1.0 0.5
f1 (ppm)

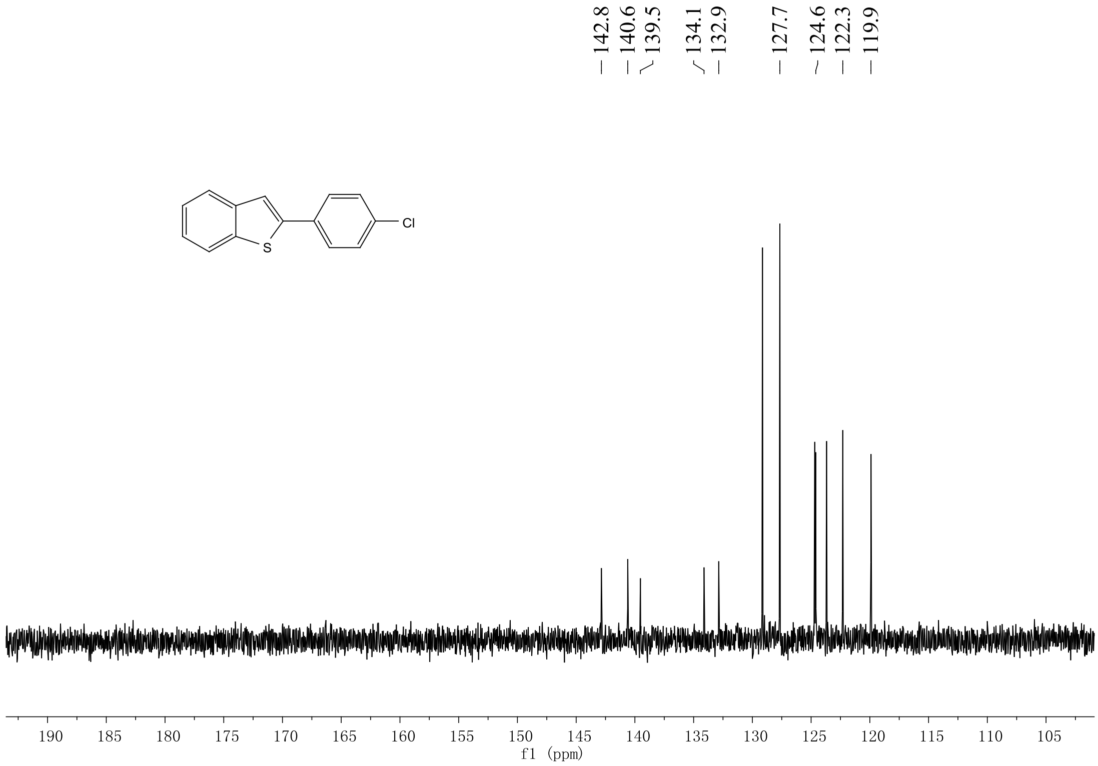
142.8
140.6
139.5
134.1
132.9
127.7
124.6
122.3
119.9
S
Cl
190 185 180 175 170 165 160 155 150 145 140 135 130 125 120 115 110 105
f1 (ppm)

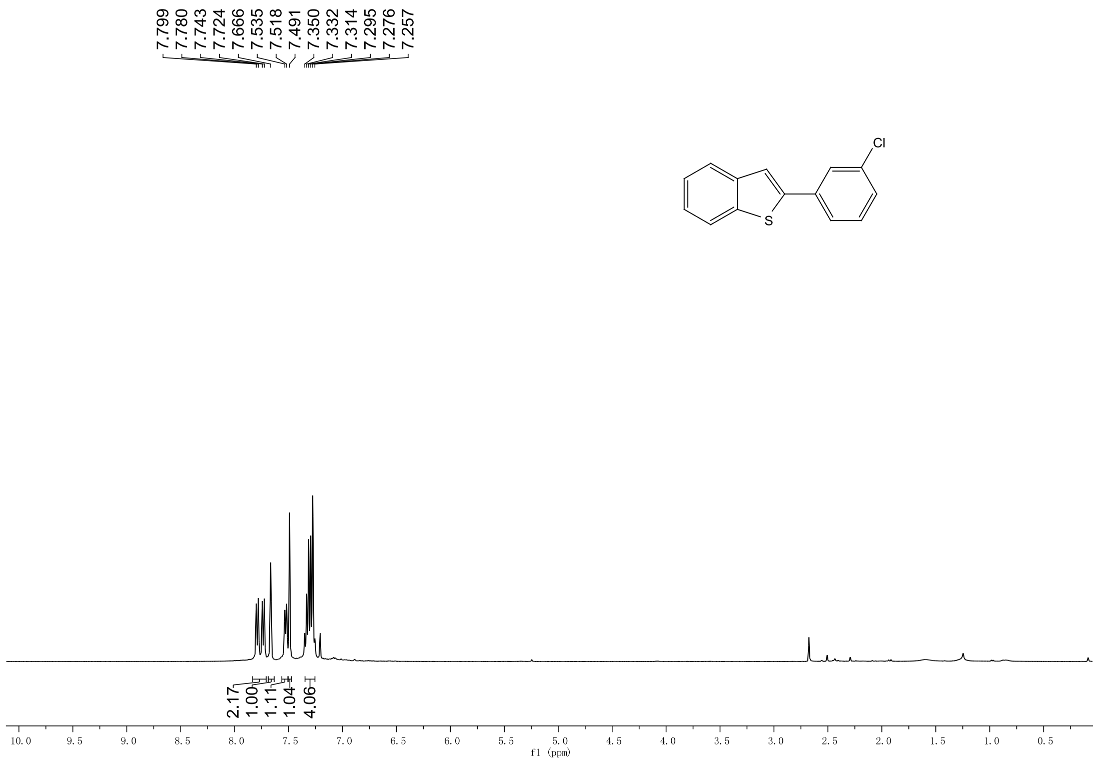
7.799
7.780
7.743
7.724
7.666
7.535
7.518
7.491
7.350
7.332
7.314
7.295
7.276
7.257
Cl
S
2.17
1.00
1.11
1.04
4.06
f1 (ppm)

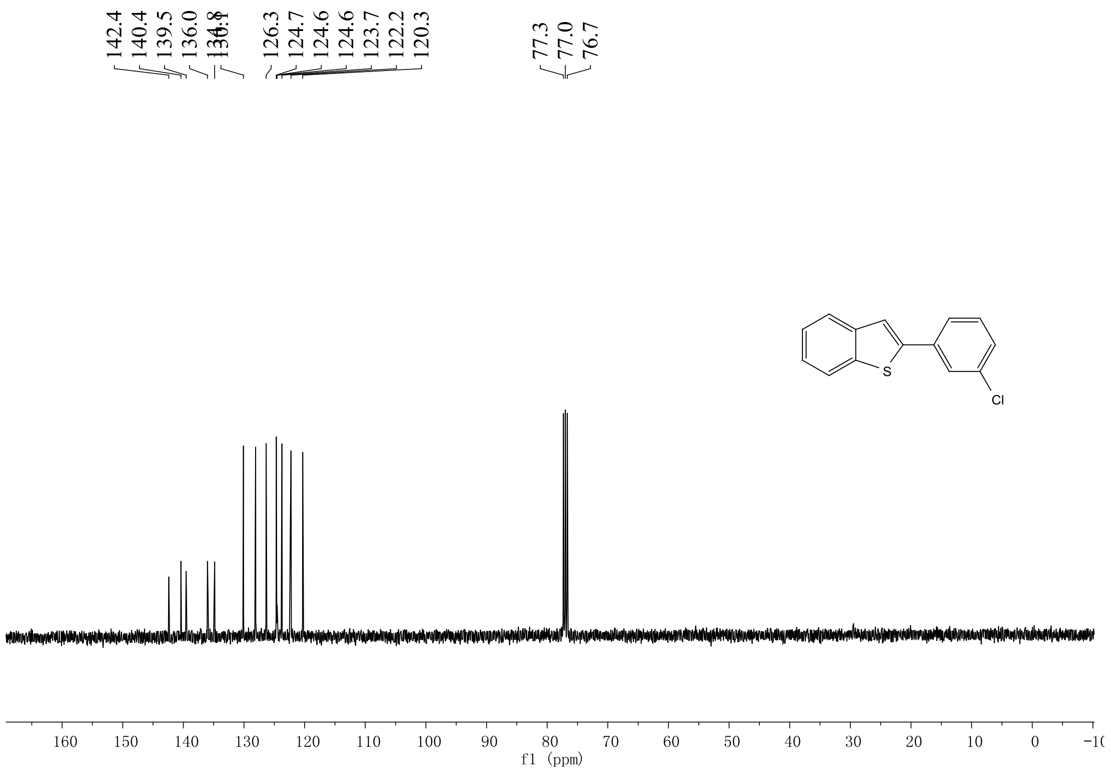
142.4
140.4
139.5
136.0
134.8
130.1
126.3
124.7
124.6
124.6
123.7
122.2
120.3
77.3
77.0
76.7
S
Cl
f1 (ppm)
